# Social identity mediates the positive effect of globalization on individual cooperation: Results from international experiments

**DOI:** 10.1371/journal.pone.0206819

**Published:** 2018-12-14

**Authors:** Gianluca Grimalda, Nancy Buchan, Marilynn Brewer

**Affiliations:** 1 Kiel Institute for the World Economy, Kiel, Germany; 2 Department of Economics, University Jaume I, Castelló de la Plana, Spain; 3 Darla Moore School of Business, University of South Carolina, Columbia, South Carolina, United States of America; 4 School of Psychology, University of New South Wales, Sydney, Australia; University of West London, UNITED KINGDOM

## Abstract

Globalization is defined for individuals as their connectivity in global networks. Social identity is conceptualized as attachment and identification with a group. We measure individual involvement with global networks and local, national, and global social identity through a questionnaire. Propensity to cooperate is measured in experiments involving local and global others. Firstly, we analyze possible determinants of global social identity. Overall, attachment to global identity is significantly lower than national and local identity, but there is a significant positive correlation between global social identity and an index of individual global connectivity. Secondly, we find a significant mediating effect of global social identity between individual global connectivity and propensity to cooperate at the global level. This is consistent with a cosmopolitan hypothesis of how participation in global networks reshapes social identity: Increased participation in global networks increases global social identity and this in turn increases propensity to cooperate with others. We also show that this model receives more support than alternative models substituting either propensity to associate with others or general generosity for individual global connectivity. We further demonstrate that more globalized individuals do not reduce contributions to local accounts while increasing contributions to global accounts, but rather are overall more generous. Finally, we find that the effect of global social identity on cooperation is significantly stronger in countries at a relatively low stage of globalization, compared to more globalized countries.

## Introduction

Globalization has been defined as the increased diffusion of worldwide connections between people [1,2]. Technological progress in various domains, from information technologies to shipping, makes it possible for people to engage with each other at unprecedented speed regardless of the distance separating them [2]. In the words of Harvey [3], globalization entails compression of time and space. This process encompasses several domains. In the economic domain, international trade and capital movements are at historically unprecedented levels. In the social domain, the internet has made possible instantaneous connections irrespective of distances. In the cultural domain, more and more people access the same sources of information or forms of entertainment worldwide. A growing awareness of the “world as a whole” [1] informs the action of many people. Indexes of globalization testify that globalization has been rising steadily over the last four decades [4,5].

The pervasiveness and comprehensiveness of globalization is likely to radically restructure individuals’ sense of the self, their social identity, their attachment to local vis-à-vis global communities, as well as their values. In spite of the relevance of this phenomenon, the empirical evidence on the issue is scant and limited to cross-country survey-based analyses. In this paper we draw on experimental evidence coming from a study that was explicitly designed to measure large-scale interconnectedness at the individual-level, and to examine its correlation with the propensity to engage in cooperative activities with global others.

It has been demonstrated that participation in global networks is significantly correlated with propensity to cooperate with global others [6–8]. More “globalized” individuals are significantly more inclined to cooperate with global others in comparison with less globalized individuals. Furthermore, the same correlation holds at the country level. The higher the aggregate level of globalization of a country, the higher the average levels of cooperation by their citizens [6].

Buchan et al. [7] show that the development of a global social identity is also positively associated with cooperation at the global level. The higher the identification with the global community, the higher one’s level of cooperation with global others. In the present paper we further expand the analysis of the linkages between globalization, social identity, and propensity to cooperate, addressing the following two questions: (1) What are the possible factors affecting global social identity? (2) Does global social identity exert a *mediating* effect in the relationship between participation in globalization and propensity to cooperate?

Our hypothesis is that participation in global networks reshapes individuals’ social identity by expanding the number and inclusiveness of groups to which individuals experience a sense of belonging and identification. In other words, we conjecture that the process of globalization expands the boundaries of the groups to which an individual attributes emotional and psychological attachment—the “ingroup”- relative to the group of people perceived as lying outside such groups–the “outgroup”. At the limit, the process of globalization may mold a *cosmopolitan* individual, for whom, as Giddens [9] suggests, “humankind becomes a ‘we’ where there are no ‘others’”.

In this paper we provide comprehensive evidence supporting what we call the cosmopolitan hypothesis [7,10,11]. We show that: (a) higher participation in global networks is associated with higher identification with the global community; (b) social identity has a mediating effect in the relationship between participation in globalization and propensity to cooperate. That is, more globalized individuals cooperate more with global others than do less globalized individuals *in as much as* their level of global social identity is higher. Both the global social identity and the individual connectivity indexes have been newly developed for our research, thus offering fresh insights into the psychological and attitudinal factors that are associated with individual propensity to cooperate. Our research was conducted in the US, Italy, Russia, Argentina, South Africa and Iran, thus spanning a broad range of the globalization spectrum and enabling us to test the generalizability of our results for countries at different levels of globalization and modernization. We conclude that the development of a “global we” identity may be one of the key elements to address problems requiring global cooperation.

## Materials and methods

### Conceptualizing globalization

Theories of globalization hint at the transcendence–or compression—of space and time in human relations as the distinctive feature of globalization. The crux of globalization is seen in the progressive elimination of physical boundaries to interpersonal relations, as a result of widespread technological progress. The range of activities that is affected by these changes is so broad that several spheres of human relations are likely to be influenced at the same time.

Even if the issue of geographical distance is certainly central to globalization, various theories differ on the emphasis they put upon it. Early definitions referred generically to “the intensification of worldwide social relations linking distant localities in such a way that local happenings are shaped by events occurring many miles away and vice versa” [9]. Other conceptualizations in turn emphasized the necessity of these links to be transnational [12], or transcontinental [13]. Other theorists [2,3] go a step further in arguing that the nature of globalization is best captured by the idea of “deterritorialization”–or “supra-territorialization”—of human relations. Scholte [14] thus discusses globalization as “the spread of transplanetary and […] supra-territorial connections between people. From this perspective, globalization involves reduction in barriers to transworld contacts. People become more able–physically, legally, culturally, and psychologically–to engage with each other in ‘one world’.”. Supra-territorialization is the characteristic that causes the spatial location of the people being connected to become irrelevant. For instance, with the internet–the supra-territorial space *par excellence–*two individuals may connect with each other regardless of their physical location, provided they have access to the network. With global trade, goods produced in any country in the world–including cultural products such as Hollywood blockbusters—can be supplied to an individual living in another country, provided that the countries are part of the international trade network. To be sure, globalization has to be understood as a *process* leading to the ideal condition of supra-territorialization, rather than as a *state* where this condition is realized under all relevant domains.

### Conceptualizing social identity

Our main conjecture is that the social, cultural, economic and psychological engagement inherent in globalization has the effect of reshaping an individual’s social identity. By social identity we mean *“that part of the individual’s self-concept which derives from his knowledge of his membership of a social group (or groups) together with the value and emotional significance attached to that membership*” [15]. Social identity relies on *categorization*–namely, the psychological process of assigning people to categories, *identification*–namely, the process whereby an individual associates him/herself with certain groups, and *comparison*–i.e. the process whereby one’s own group is compared with other groups [16]. A key distinction is put forward between the “ingroup” and the residual category of the “outgroup”. An ingroup can be defined as a group to which an individual (a) categorizes herself as being part of, (b) identifies with, and (c) triggers comparisons with other groups.

Turner et al. [17] proposed three possible levels of self-categorization, categorization at the level of humankind being the highest. At the intermediate level differences between one’s ingroup and outgroup and similarities within one’s ingroup help define the self, while at the lowest level it is the differentiation from other ingroup members that shapes an individual’s identity. Most of the research effort related to social identity has thus far focused on the intermediate level of ingroup-outgroup categorization, investigating the conditions under which ‘ingroup favouritism’, i.e. a tendency to treat more favorably ingroup members than outgroup members in situations of strategic interaction, is generated [18–26]. Little attention has been devoted to the exploration of the highest level of self-categorization [10,11,27]. This paper aims to contribute to fill this gap.

### The link between globalization and social identity

Theories of globalization suggest opposite ‘ideal types” that result from the process of globalization, namely, the “cosmopolitan” individual and the “reactant” individual [6,28]. The former suggests that individuals involved in global networks experience heightened global social identity. The ingroup boundary is shifted outward to include groups of people formerly conceived as part of the ‘outgroup’. At the limit, this process may involve the whole of humanity [9,13,17,29]. The flourishing of several ‘global’ social movements around a variety of causes such as human rights or the environment, and the growing importance of global humanitarian relief operations are all instances of the diffusion of a ‘cosmopolitan’ individual [30,31].

In contrast, the “reactant” individual hypothesis predicts increased attachment to traditional loyalties, such as local and national communities, as an effect of globalization. According to this model, globalization enhances even further the cleavage between ingroup and outgroup [28,32,33], as it triggers a negative reaction by the individual against the global flows of objects, commodities, people, ideas. This may lead to an entrenchment in the state-nation community or even to adhesion to fundamentalist movements [28,34]. In terms of the ingroup-outgroup model, the presence of an “other” is made more vivid to members of an ingroup, thus strengthening even further the constricted parochial boundary between the “us” and “them”.

Buchan et al. [6,7] found evidence consistent with the “cosmopolitan” ideal-type. Individuals who were more involved in global networks were significantly more inclined to cooperate with global others than individuals who were less globalized [6]. Identification with the “world as a whole”, that is, the distinct notion of the common fate shared by many individuals around the globe as a result of their increased inter-connectedness [1] is also an important aspect of globalization. Individuals who identify most with the “world as a whole” relative to national and local communities are more inclined to cooperate with global others [7].

Importantly, these results suggested a transformation of motives and values from self-interest to group interest and concern for the welfare of the group such that increases in global social identity are associated with increased cooperation with the global collective. Significantly, this positive effect of global social identity on cooperation was above and beyond expectations about how others in the group would behave.

### Direct and indirect effects of involvement with globalization and propensity to cooperate: The cosmopolitan hypothesis

Building on our understanding of the linkages between globalization, social identity and cooperation just presented we argue that participation in global networks may both have a *direct* and an *indirect* effect on cooperation. We classify as *direct* effects of individual involvement with global networks all those effects that take place independently from the restructuring of global social identity. Such direct effects may occur for a variety of reasons. Increased involvement in global networks may increase the amount of information and knowledge that an individual has about people living outside local and national communities. Global networks provide individuals with information about events taking place in far-away places, report on global-others’ life-style and cultural traits and distribute products and objects from foreign countries. The idea of a “global other” may thus turn from being a remote and indefinite notion to a more concrete and well-defined image of geographically distant people living in a globalized world. Such increased familiarity with groups of people previously held as remote–both in geographical and social terms–may trigger increased propensity to cooperate.

Increased involvement in global networks may also make an individual more aware of the opportunities arising from cooperating worldwide. Deeper awareness of the global nature of the problems facing people from all around the world may instill a greater consciousness of the importance of global cooperation and may increase the symmetrical expectation that global others also become more conscious about the necessity of global action. This increased awareness in itself may strengthen the propensity to cooperate at the global level. Moreover, the observation of cases in which global others have successfully achieved and maintained cooperation may increase an individual’s trust in them, thus strengthening a positive disposition to cooperate.

**Fig 1:**
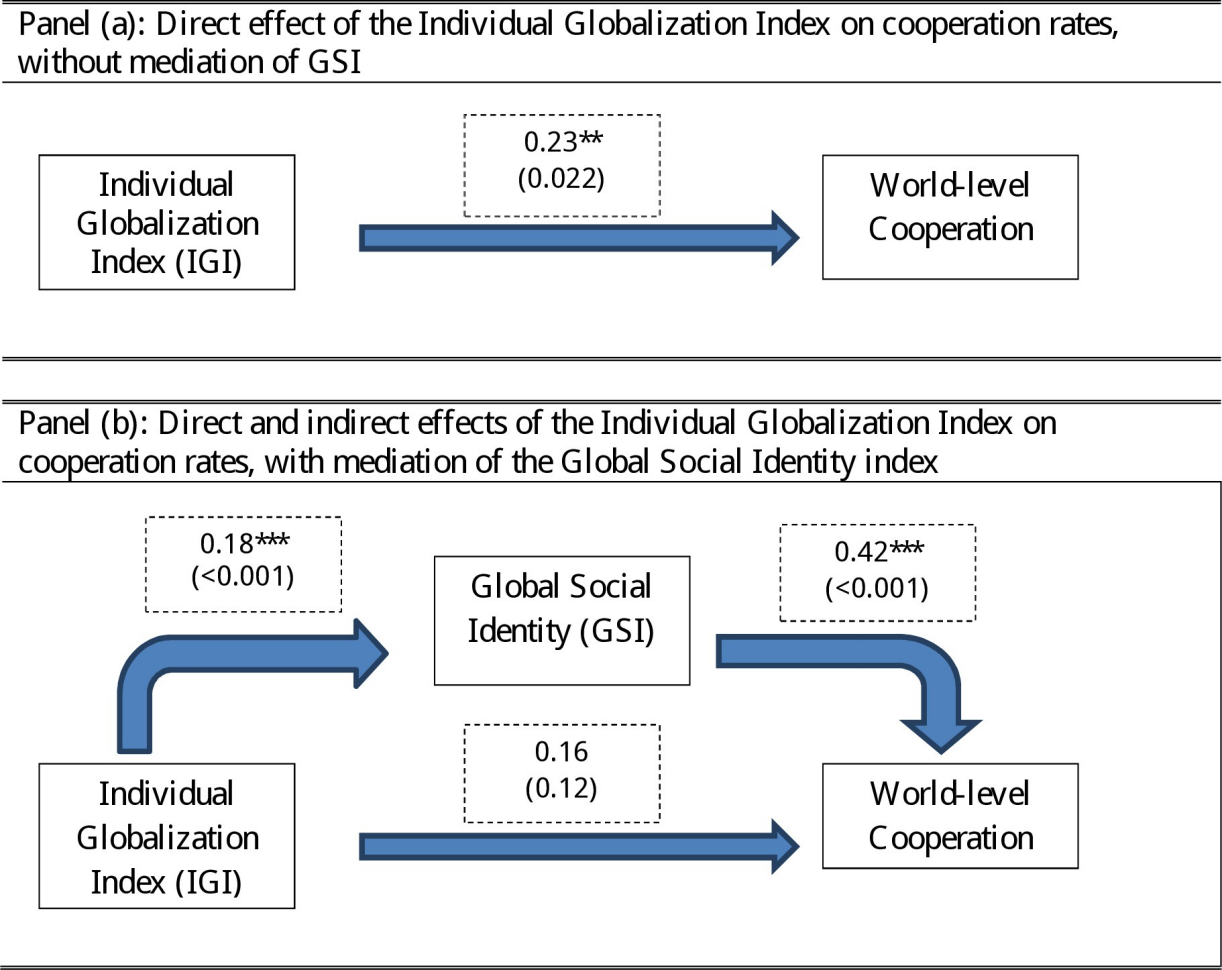
Mediating effect of global social identity between individual involvement with globalization, as measured by the IGI, and propensity to cooperate at global level. The values in the dashed-contour boxes are the coefficients, expressed in units of standard deviation, estimated in an OLS econometric analysis using the same models as in Table 2. The number in parenthesis is the p-value of the test that the coefficient is equal to zero. The stars denote the level of significance of the rejection of the null hypothesis (* = p<0.1; ** = p<0.05; *** = p<0.01). Panel (a) reports coefficients for the model that does not include GSI as covariate (corresponding to Table 2: column 1); Panel (b) reports coefficients for the models including GSI as covariate (corresponding to Table 2: column 2 and 3). Fig 1 reports coefficients based on the OLS estimation that has been used for the Sobel-Goodman mediation test reported in Table 3, column 1.

In addition to these mechanisms, we also put forward what we refer to as the “cosmopolitan hypothesis”. We posit that participation in global networks may have an *indirect* effect on cooperation with global others, inasmuch as it increases one’s identification with the global community. Such a mechanism can be broken down into two constitutive parts. Firstly, increased participation and involvement in global networks bring about heightened identification and attachment to the global community. More individuals will find the global community as being a relevant part for the construal of the self, and they will do so with higher intensity. As a result, global social identity increases. Secondly, social identity theory argues that increased identification with a group goes hand-in-hand with increased propensity to cooperate with that group [16,17,35–40]. When individuals attach their sense of self to their group membership, they see themselves as interchangeable components of a larger social unit [17]. This engenders a shift of motives and values from self-interest to group interest and concern for the welfare of fellow group members. Pursuing the group’s interest thus becomes a direct and natural expression of self-interest. When these two constitutive elements operate together, increased involvement with the global networks will increase identification with the global community, and this in turn will be accompanied by increased propensity to cooperate with global others. This mechanism is visually illustrated in Fig 1B.

### The experimental measure of cooperation

Our project involved adult populations from specific locations in six different countries (Iran, South Africa, Argentina, Russian Federations, Italy, and the US). Participants in our research took part in three experimental decisions that assessed their propensity to cooperate in Public Goods Games (PGG). No feedback was given at the end of each decision, thus successive choices could not be influenced by the outcomes of prior decisions. Here we discuss the last of the three decisions, which entailed cooperation at the global level. Cooperation was measured through a Multi-level Sequential Contribution (MSC) game. The setting is similar to standard PGGs except that participants’ decisions were made sequentially rather than simultaneously. Participants’ decisions affected the payoffs of other participants taking part in future sessions. In turn, participants’ payoffs were determined by their own decisions, as well as by decisions made by participants in previous sessions. Details of the experimental procedures can be found in the S4 Appendix.

Each participant was endowed with 10 tokens, each worth the purchasing power equivalent of US $0.50 in each country. In the third experimental decision, participants made their decisions by allocating the 10 tokens across three different envelopes, named “Personal”, “Local”, and “World”. Each token allocated to the Personal account was simply transferred to the participant’s final earnings account and yielded no benefits to others. That is, its Marginal Per Capita Return (MPCR) was one. Conversely, each token allocated into the Local envelope was added to the Local contributions by three other participants from the same locality. This total was doubled by the experimenter and the participant received one-quarter of the total. Thus, each token allocated to the Local account entailed a half token loss for the participant and yielded a half token gain to three other participants from the same local area. The MPCR for the local account was 0.5 (less than the MPCR of the Personal account), but the Marginal Social Return (MSR), measuring the returns for the group, was 2.

The World account comprised the participant, the same three local people who were part of the Local account, and two four-person groups from two different countries. The specific countries were not named. Rather, participants were informed that these countries might have been from any of the four continents where the research was conducted. Not naming countries made choices unaffected by biases or stereotypes about particular nationalities. This is important because stereotypes can be deeply enrooted and widespread worldwide, while being at the same time fundamentally wrong [41]. This approach is also consistent with our definition of globality as a notion that transcends mere internationalization. Tokens allocated to the World account were summed, tripled by the researcher and the participant received a one-twelfth share from the total. Each token allocated to the World account thus yielded a ¾ token *loss* for the subject and yielded a ¼ token *gain* to each of the 11 others matched with that subject. The MPCR is 0.25 and the MSR is 3 for the World account. Thus, contributing to either the Local or World account can be classified as a cooperative act in that the individual sacrifices immediate personal gain for greater gain at the collective level. Participants’ identity was not revealed either to other participants or to the experimenter, as the game was played in conditions of anonymity. Participants were told that they were involved in a series of decisions involving people from their own local area, some of whom may or may not be in the same room, and from other countries around the world.

**Fig 2:**
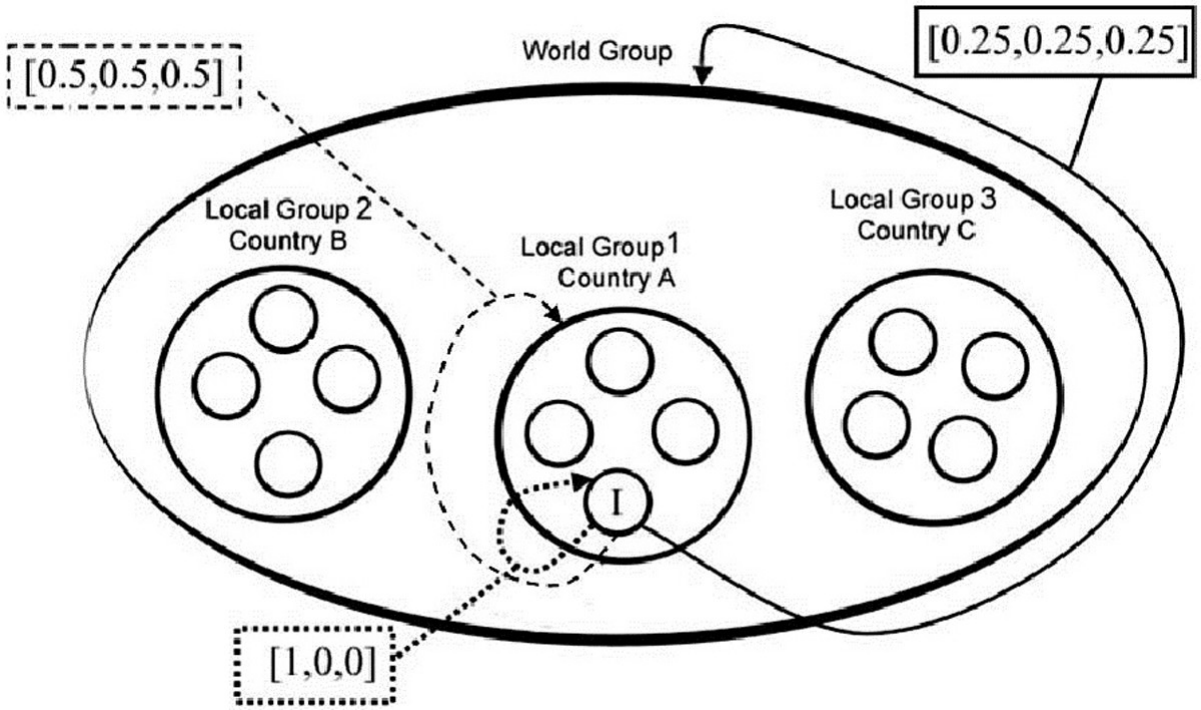
Representation of the nested social dilemma. I stands for “Individual”. ‘Local 1’, ‘Local 2’, ‘Local 3’ represent groups of people residents in the same locality in three different countries. Individuals have three options on how to allocate their endowments of 10 tokens: allocating to a personal account, to their local account, and to the global account, which comprises the three lower-level local accounts. Contributions to the personal account are transferred one-to-one onto an individual’s payoff. Contributions to one’s local account are multiplied by a factor of two and divided among four local residents. Contributions to the global accounts are multiplied by a factor of three and divided evenly among the 12 participants.

The structure of incentives resembled a nested PGG similar to that employed by Blackwell and McKee [42] and Wit and Kerr [43]. The design is seen schematically in Fig 2. In the MSC, an individual willing to maximize her final payoffs should allocate all tokens to the Personal account, because both the Local and World accounts bear a smaller MPCR. If no one contributed, each participant would take home their initial 10 tokens. Prior research shows that many individuals choose to act in the interests of the group [44]. In our MSC there is a tension between individual returns, social returns, and the locality of the people benefitting from one’s contribution. Individuals allotting their tokens to their Local account can so ensure the maximization of the interests of the Local constituency. But if everyone contributed their endowment to their Local account, the final individual payoffs would be 20 tokens, which is less than if everyone allotted their tokens to the World account, that is, 30 tokens. We regard contributions to the Local account as being driven by parochial interests, whilst contributions to the World account reflect cosmopolitan interests. In the remainder of the paper we will refer to them as Local-level and World-level cooperation, respectively.

The second experimental decision, which we do not analyze in this paper, was another MSC where a National account replaced the World account, as people interacted with other groups from their country but not from their locality. The first experimental decision was a standard linear PGG involving only people from the same locality as the group. We use cooperation in the first decision as a control for the baseline propensity to cooperate at the local level in the foregoing analysis.

Participants’ decisions were collected by the researchers and randomly matched with previous participants’ decisions through an electronic algorithm. Payments were calculated while subjects completed a questionnaire and were handed out at the end of the session. Average take-home earnings from the experiment were the purchasing power equivalent of US$34, including the purchasing power equivalent of a US$8 show-up fee. Sessions lasted around 90 minutes. Our MSC design yielded three important features. First, this design realistically mapped onto the nature of local-global relations. In the global economy, globalization does not exclude the local constituency but potentially expands the level of inclusion to both local and non-local participants. Second, our design also captured the tension between the different incentives from giving to the local vis-à-vis the global good. In our design, the MPCR from giving to the local public account is greater than that of the global account; but on the other hand, the social return is higher in the latter. In this fashion, we are able to examine under which conditions individuals put global interests ahead of local ones when everyone might be able to benefit in the long run. Third, our design was as parsimonious and easily-understood by participants as possible. Preliminary tests of different versions of the games on college students in the US, Canada, and Spain, demonstrated that the return ratios we adopted was the most easily understood by participants.

### Questionnaire-based variables

The main dependent variable of our analysis -namely, the individual’s allocation to a global public good in a nested PGG—was obtained from the MSC just described. The independent variables for our analysis come from an individual level questionnaire that participants completed at the end of the experiment.

The first and most important aspect that the questionnaire was designed to measure was individual exposure and participation in global relations. This measure, originally developed for our research, is–to the best of our knowledge—the first example of an individual level index of globalization. Analogous to the country-level globalization index (CGI) developed by the Center for the Study of Globalisation and Regionalisation [4] (see Tables A and B in S1 Appendix), the questionnaire was designed to capture individual access to globalization within the social, cultural, political, and economic spheres. The resulting Individual Globalization Index (IGI) is a summative scale of 30 questionnaire items listed in Table C in the S1 Appendix. The text of the questions is reported in the S3 Appendix. Further methodological details on the construction of both the CGI and the IGI are illustrated in the S2 Appendix. The IGI measures an individual’s usage of various global networks in terms of two dimensions: the frequency with which an individual accesses the networks, and the territorial scope. The index identifies several media of global connection and measures the temporal frequency with which the medium of connection is used by the individual and whether such a medium is used to contact people at the local, national, or global level. Although a given medium of connection, such as the email, has a potentially global reach, an individual can also decide to use it for contacts at the local or national levels. The IGI, therefore, assigns higher scores to individuals who participate in the global network more frequently and on a larger scope than others.

In addition to the IGI items, a set of three social identity measures was included in the questionnaire. The items were taken from the measure of social identity constructed by Yuki et al. [45] and adapted to assess social identification at the levels of the local community, the nation, and the world. For example, in Kazan, Russia, the items measuring social identity at the level of the local community read:

1. How strongly do you feel attachment to *your community in Kazan*?
2. How strongly do you define yourself as a member of *your community in Kazan*?
3. How close do you feel to other members *of your community in Kazan*?

Social identities at the national and global level are measured substituting the following expressions, respectively, for “your community in Kazan”: *“your community in Russia”*, and *“the world as a whole”*. Responses to each item are made on a rating scale from 1 (not at all) to 4 (very much).

The questionnaire also included some questions to assess awareness of, and attitudes toward global processes. Robertson [1] suggests that a key aspect of globalization is, in addition to participation into global networks, the “consciousness of the world as a whole”. It is therefore important to assess how the key constructs in our analysis relate to one’s global awareness. We constructed a ‘Global Awareness Index’, based on the answers to four questionnaire items inquiring about a participant’s awareness of the following global issues: global warming, the spread across the planet of potentially dangerous diseases, the action of the International Criminal Courts of justice, and the persistent gap between rich and poor people around the world. Other questions measured an individual’s attitudes towards global processes. Some, taken from the World Value Survey [46], were included to measure the presence of ethnocentric attitudes, specifically, the participant’s willingness to restrict migrants’ access, and the necessity to protect national culture from foreign influence. Other questions from the PEW [47] Global Attitudes Survey inquired about a participant’s opinions on international trade and migration. Finally, standard demographic measures were included to control for factors such as age, gender, level of income, ethnicity, education, and employment. Descriptive statistics for the main variables of interest, for the demographics of the sample are reported in Tables D and E in S1 Appendix.

### Selection of research environments, sampling techniques, and implementation

Research sites were selected for this research with the goal of representing a sufficient degree of variability on the globalization spectrum as ranked by the CGI [4]. Six countries were chosen, with the aim of both maximizing the dispersion of each sphere of the CGI–namely, the economic, social, and political sphere–and of ensuring a sufficient geographic dispersion, so that each continent–apart from Oceania–was represented. The choice fell on Italy and Argentina (respectively, at the highest and lowest positions in the economic globalization sub-index); US and South Africa (at the extremes of the social globalization index); Russia and Iran (at the extremes of the political globalization index).

We selected several locations in each country which, on the basis of available information prior to conducting research, represented differing levels of exposure to globalization as per, for instance, the relative presence of multi-national corporations or the presence of immigrant populations. In general, in each country a large urban center was designated as the ‘hub’ of the fieldwork, and less globalized towns or villages were selected within a radius of around 100 miles. Hub localities in the US, Italy, Russian Federation and Argentina were Columbus (Ohio), Milan, Kazan (Tatarastan), and Buenos Aires, respectively. For logistical constraints, the same strategy was not feasible neither in Iran nor South Africa. In Iran the two research sites were Tehran–Iran’s capital and largest city–and Shiraz–the fifth largest city [48]. In South Africa research sites were three districts of Northern Johannesburg and the district of Soweto, residents of the latter district being almost exclusively of Black ethnic background. Research sites within Iran and South Africa are nonetheless characterized by appreciably different degrees of exposure to globalization within each country, thus ensuring the comparability of our samples across countries. Our econometric analysis includes country fixed effect, thus ensuring that any difference in the sampling strategy across countries is controlled for.

Approximately 200 participants were recruited in each country according to a quota sampling method. The quota sampling method aims to target a uniform distribution of observations across relevant demographic dimensions. This method is suitable for cross-country comparative research because it achieves comparability. In our study, the criteria determining the quotas were age (three categories: 19–30, 31–50, 51–70), gender (two categories: male, female), and social economic status (three categories: high, intermediate, and low).

The Institutional Review Board (IRB) of the University of Wisconsin-Madison and the Ethics Committee of Warwick University provided ethical approval to the project. Oral informed consent was obtained from every participant. The opportunity of obtaining written consent from participants was discussed with the University of Wisconsin-Madison IRB. The IRB finally requested oral consent rather than written consent. The IRB feared that keeping a written record of participants’ names may have possibly compromised their safety in countries such as Iran and Russia, because of the risk that participants would be associated with pro-US activities, given the nationality of the project’s main funding body–the US National Science Foundation. A template of the information and informed consent form and of instructions are reported in the S4 Appendix.

## Results

### Descriptive statistics

The social identification scores at each level (local social identity—LSI; national social identity—NSI; and global social identity—GSI) were calculated by summing up responses to the three items. The scores, given originally in a 1–4 scale, have been normalized to the 0–1 interval. So, individuals scoring one (zero) in, say, the LSI answered that they feel very strong attachment (no attachment) to their local community, define themselves very strongly (not at all) as a member of their local community, and feel very close (not close at all) to other members of their local community. The Cronbach’s alphas of the three social identity items are 0.78 for LSI, 0.72 for NSI, and 0.75 for GSI.

**Fig 3:**
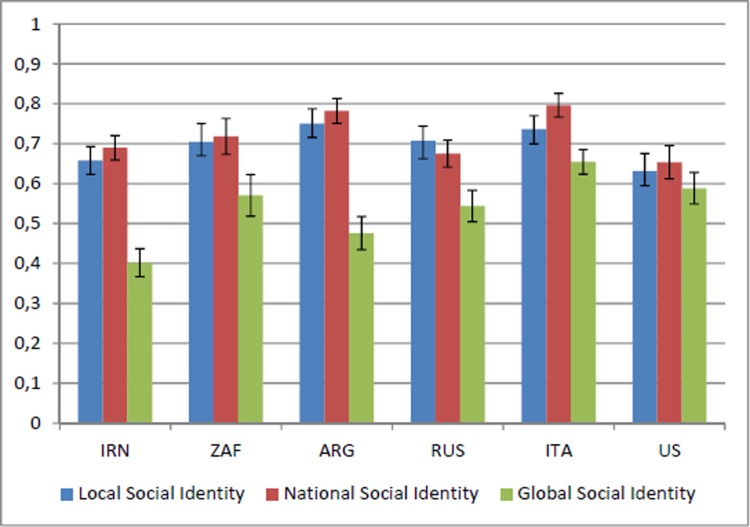
Average levels of local, national, and global social identity, per country. Descriptive statistics for the whole sample are reported in Table E in the S1 Appendix.

Fig 3 reports the average values of the three social identity measures in each country. For all countries, except the Russian Federation, the strongest identification occurs on average at the national level, followed by the local and then the global level. In the Russian Federation, identification is strongest at the local level, followed by the national and the global level. According to non-parametric Wilcoxon sign-rank tests, the difference between LSI and NSI is not significant in any country except for the Russian Federation (p<0.001; see Table F, panel d in the S1 Appendix) – where participants tend to report higher LSI than NSI–and Italy (p<0.001; see Table F, panel e in the S1 Appendix) – where on the contrary participants report higher NSI than LSI. The result in Russia may be driven by the ethnic diversity of the sample, as people with ethnicity different from Russian identify more with local than national identity. Merging all observations together, there is no statistically significant difference between LSI and NSI (p = 0.20; Table F, panel g in the S1 Appendix). On the contrary, the difference between GSI and the other two social identity measures is always statistically significant (see Table F, panel g in the S1 Appendix). The country where such differences are relatively contained and do not always reach strongly significant differences is the US (p = 0.070 for the difference between LSI and GSI; p = 0.0026 for the difference between NSI and GSI; see Table F, panel f in the S1 Appendix).

McFarland et al. [10] developed a measure of “Identification with all humanity” (IWAH) that evaluates the extent to which an individual “cares for all humanity, not just for their ingroups”. The general structure of the IWAH measure is similar to our social identity indexes, because respondents are asked to evaluate their identification with, and attitudes toward, (a) people in their community, (b) co-nationals, and (c) “all humans everywhere” [10]. Although the phrasing used to identify these three categories differs slightly from the one we used, the two measures appear comparable. In a sample comprising US participants only, the IWAH measure records the same pattern we found in our study, with identification with global community being lower than identification with local and national communities, the latter two being approximately equal to each other. Our analysis reported above enables us to say that this same pattern holds, even more pronouncedly, in other countries, being the US at the lower end of the differences between GSI and the other social identity measures.

### Analysis of the factors associated with GSI

An implication of the cosmopolitan model of social identity is that increased participation and exposure to global networks should be associated with increased identification with the global community. We test for this idea through a tobit model. The first specification (see Table 1, column 1) demonstrates a strongly significant correlation between GSI and both CGI and IGI. That is, people living in more globalized countries and those who are more involved in global networks are also more likely to declare higher identification with the global community. In other words, the more an individual participates in the global network, the higher their GSI.

**Table 1:** Regression analysis of factors associated with GSI.

DEPENDENT VARIABLE	GSI			
	(1)	(2)	(3)	(4)
CGI	0.217***	0.264***		
	(0.0468)	(0.0417)		
IGI	0.564***	0.521***	0.487***	0.287***
	(0.0916)	(0.0830)	(0.0865)	(0.0866)
National Social Identity		0.492***	0.497***	0.508***
Index		(0.0481)	(0.0492)	(0.0496)
Local Social Identity Index		0.110**	0.106**	0.0741*
		(0.0450)	(0.0450)	(0.0445)
Foreign Immigrants	-0.00357	-0.0159	-0.0152	-0.0238
	(0.0271)	(0.0243)	(0.0242)	(0.0240)
Female	0.0637***	0.0645***	0.0605***	0.0538***
	(0.0199)	(0.0178)	(0.0182)	(0.0179)
Education Medium	0.0568**	0.0393	0.0254	0.0260
	(0.0262)	(0.0239)	(0.0255)	(0.0247)
Education High	0.0370	0.0388*	0.0354	0.0256
	(0.0239)	(0.0213)	(0.0225)	(0.0222)
Age Medium	0.0244	-0.0178	-0.00986	-0.00836
	(0.0230)	(0.0206)	(0.0205)	(0.0204)
Age High	0.115***	0.0475**	0.0487**	0.0363
	(0.0263)	(0.0239)	(0.0237)	(0.0245)
Income Medium	-0.0273	-0.0249	-0.0241	-0.0149
	(0.0251)	(0.0224)	(0.0224)	(0.0222)
Income High	-0.0833***	-0.0933***	-0.0895***	-0.0671**
	(0.0299)	(0.0265)	(0.0283)	(0.0291)
City	-0.0158	-0.0281	-0.0339*	-0.0149
	(0.0223)	(0.0199)	(0.0203)	(0.0200)
Global Awareness Index				0.245***
				(0.0462)
Association Membership				0.0447**
				(0.0200)
Way of Life				-0.0327***
				(0.00983)
Entry				-0.0265***
				(0.00931)
Opinion Glob.				-0.0177**
				(0.00823)
Self Employed				0.0529
				(0.0329)
Unemployed				0.0361
				(0.0506)
Divorced				-0.00859
				(0.0278)
South Africa			0.128***	0.105**
			(0.0384)	(0.0415)
Argentina			0.0176	-0.0187
			(0.0361)	(0.0372)
Russia			0.134***	0.124***
			(0.0307)	(0.0303)
Italy			0.169***	0.174***
			(0.0331)	(0.0327)
USA			0.172***	0.131***
			(0.0330)	(0.0342)
Constant	0.156***	-0.226***	-0.173***	-0.0867
	(0.0505)	(0.0519)	(0.0506)	(0.0670)
Observations	998	994	994	948
Pseudo R^2^	0.133	0.366	0.385	0.482

**Note**: A tobit model has been fitted to the data. The censoring values are the lowest and upper values for GSI, i.e. 0 and 1. Robust standard errors are in parentheses.

*** p<0.01,

** p<0.05,

* p<0.1.

The description of variables is in Table E in the S1 Appendix.

Among the demographic factors, females and people older than 50 years (variable ‘Age High’), are also more likely to score high in GSI. Having attained higher levels of education than the primary level (variable ‘Education High’) also shows a positive effect on GSI, but this is not robust to the inclusion of further controls in the ensuing regressions. Interestingly enough, the variable ‘Income High’, identifying people reporting a level of income belonging to the seventh, or upper, decile of a country’s income distribution has a significantly negative effect on GSI (p = 0.005), in relation to people with low income (lower or equal to the third decile). We further investigate this result below. Living in large urban areas (variable ‘City’) or in areas with relatively high numbers of foreign immigrants (variable ‘Foreign Immigrants’) seems to be uncorrelated with GSI.

The second model (see Table 1, column 2) includes both NSI and LSI as controls. An individual may experience attachment to *any* group, rather than experience specific attachment to the global community. In this second specification, the results are to be understood as analyzing the impact of a variable on GSI *relative to* LSI and NSI. Both LSI and, even more so, NSI show positive correlations with GSI. A one standard deviation unit in NSI increases GSI by 0.42 standard deviation units (p<0.001), while the impact for LSI is smaller, namely, 0.11 (p = 0.015). Both CGI and IGI continue to exert a positive and strongly significant effect on GSI. The same holds for gender and high income (p<0.001 for all these four variables). Females’ GSI scores are, *ceteris paribus*, nearly 6% higher than men’s scores. This result goes hand-in-hand with females scoring higher in our Global Awareness Index (p = 0.011). Conversely, McFarland et al. [10] find no significant effect of gender and greater knowledge of global issues by males rather than females. The effect of belonging to the older age group is also still significant (p = 0.047). The positive correlation between age and GSI may be surprising, in the light of the emphasis posed by some scholars on younger generations being particularly exposed to the influence of global culture [28]. Nevertheless, we note that the IWAH scale developed by McFarland et al. [10] also found lower identification with *any* of the three categorization levels (local, national, and global) in a university student sample than in an adult sample, thus indirectly confirming our result.

These first analyses are “between-country” because of the omission of country dummies. This may introduce some confounding effects if some variable is correlated with country-level globalization. For this reason, regression 3 introduces country dummies so the analysis is now to be understood as being “within-country”. The introduction of country fixed effects obliterates from the analysis all variables that are invariant within-country, such as CGI. In results from this third regression analysis, IGI (p<0.001), NSI (p<0.001), and LSI (p = 0.019) maintain strong positive effects, as well as gender (p = 0.01), higher age (p = 0.040), and higher income (p = 0.002) (see Table 1, column 3).

The last specification (see Table 1, column 4) includes several additional variables measuring a participant’s ‘Global Awareness Index’ (see “Materials and Methods”), some attitudinal measures concerning globalization, and variables identifying the participant’s occupational situation. All these variables are derived from the questionnaire, as described in Table E in the S1 Appendix and in the S3 Appendix. The regression shows that people who are more aware of global issues report significantly higher scores for GSI (variable ‘Global Awareness Index’) (p<0.001). McFarland et al. [10], too, found a high correlation between their IWAH and both global knowledge and global humanitarian concerns.

Other attitudinal measures are also significantly related with the GSI. The less a participant believes that their citizens’ way of life needs to be protected against foreign influence (variable ‘Way of Life’), and that entry of foreigners should be restricted (variable ‘Entry’), the higher their GSI (p<0.01 for both variables). These results are again in line with McFarland et al. [10], who found a strong predictive negative power of their measure of ethnocentrism and their IWAH. Additionally, we find that the more the participant believes that trade, global business, faster communication and greater movements of people are a good thing (variable ‘Opinion Glob.’), the higher their GSI score (p = 0.015). It is also noteworthy that participants scoring high in GSI are significantly more likely to be active in voluntary associations (variable ‘Association Membership’) (p = 0.026).

Among the demographic controls, gender (p = 0.003) and high income (p = 0.021) continue to exert significant effects, while belonging to the older age group becomes non-significant (p = 0.138). The apparent robustness of the effect of ‘Income High’ warrants further investigation. We note that ‘Income High’ is highly correlated with IGI (*ρ* = 0.39) and we suspect that this may cause multi-collinearity problems. In fact, when IGI is omitted from the model, ‘Income High’ is no longer significant (p = 0.207). We also note that the raw linear correlation between ‘Income High’ and GSI is relatively low (*ρ* = 0.03), while the correlation between IGI and GSI is considerably larger (*ρ* = 0.19). We compute the Variance Inflation Factor (a measure of how much a variable may create multi-collinearity problems in a regression) for ‘Income High’. Such a factor is very close to the threshold suggested by Allison [49] to signal serious multi-collinearity problems (2.45 vis-à-vis a suggested threshold of 2.5), and, except country dummies, is the variable contributing the most to inflating variance. We conclude that the negative sign of ‘Income High’ appears to be driven by its correlation with IGI rather than signaling a real independent effect.

The occupational variables are not significant, although ‘Self-employed’ is at the border of significance (p = 0.109). Finally, the IGI maintains a strongly positive effect on GSI (p = 0.001), even after all these demographic and attitudinal variables are controlled for. This further proves the robustness of the correlation between participation in the global network and GSI.

We conclude:

**Result 1**: Consistent with the cosmopolitan ideal-type, increased participation in global networks–both at the individual and country levels—is associated with increased identification with the global community.
**Result 2**: The analysis of several attitudinal factors confirms the validity of the GSI construct. Generally speaking, individuals reporting high GSI scores express a positive view regarding global flows of people and objects, and are more aware of global issues than individuals who have lower scores. Women and, although less robustly, older people and more highly-educated people report higher GSI scores. Income is negatively related with GSI, although this result is likely to be driven by the strong correlation between income and IGI.

### Analysis of the mediating effects of GSI between participation in global networks and cooperation levels

In this section we investigate the relationships between IGI, GSI, and World-level cooperation, examining whether GSI may be thought of as having a mediating effect on IGI as per the cosmopolitan hypothesis.

**Fig 4:**
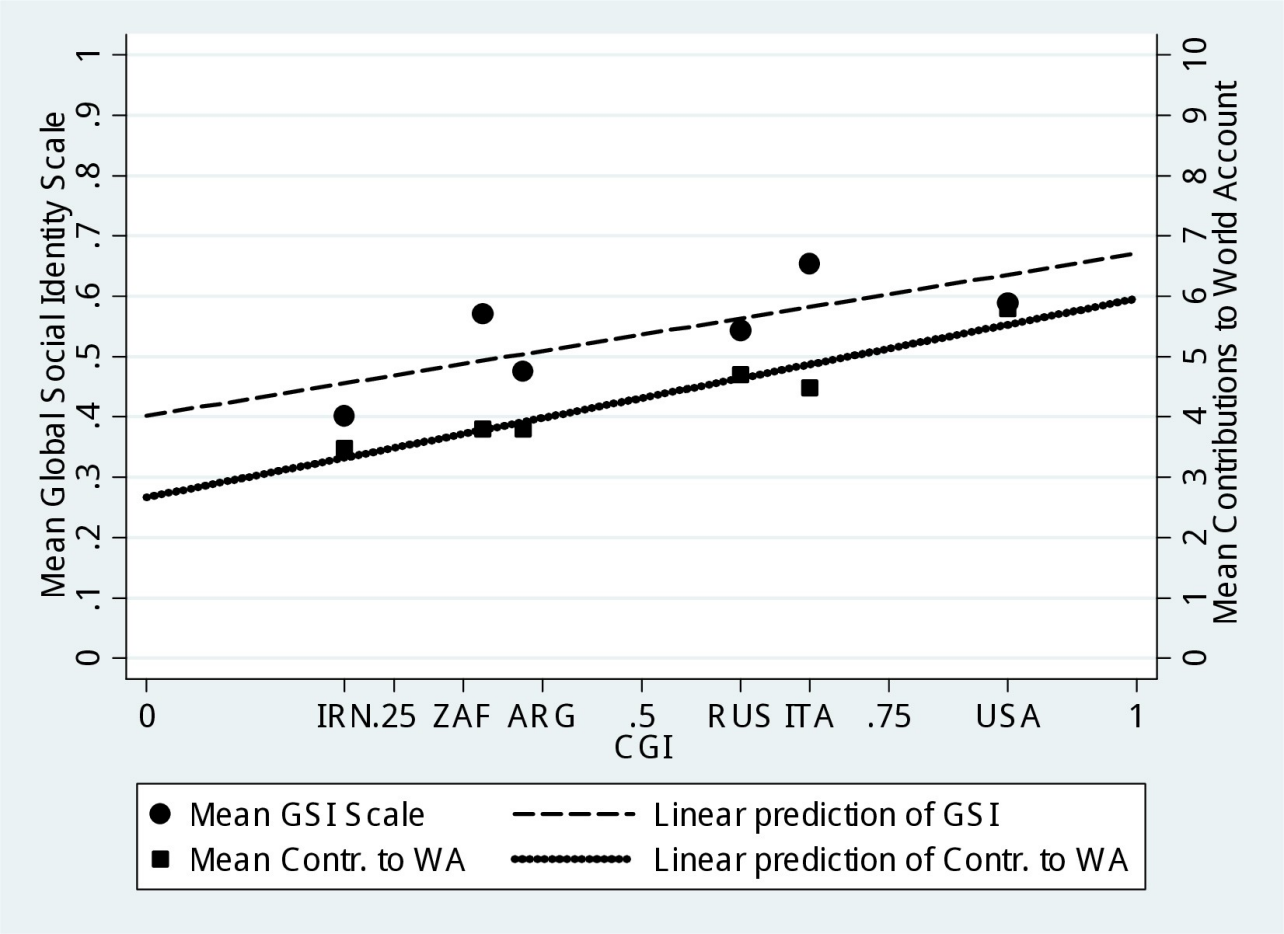
Correlation between country-level globalization index, global social identity scale, and world-level cooperation. The chart reports country-level mean values for the GSI index (circles), and the contribution to world account (WA) (squares), as well as the linear predictions of GCI onto GSI and contributions to WA (dashed and solid lines, respectively).

Fig 4 offers a graphical account of the relationship between CGI, GSI and World-level cooperation at the country level. It plots the mean level of both GSI and World-level cooperation, as a function of the country’s CGI. A linear prediction of each variable shows a positive relationship. This means that the more a country is globalized, the more participants from that country score high on the GSI and the more, on average, they contribute to the world account.

We perform a Sobel-Goodmann test [50] on the hypothesis that GSI exerts a mediating effect between IGI and World-level cooperation. The main idea behind this test is that for a variable *z* exerting a mediating effect between two variables *x* and *y*, the following three conditions must hold: (1) *x* significantly influences *y* in the absence of *z*; (2) *x* significantly influences *z*; (3) Once z is introduced as a covariate alongside *x*, the effect of *x* shrinks considerably, while *z* exerts a significant effect on *y*. In the first specification, we show that condition (1) holds (see Table 2, column 1). That is, IGI exerts a positive effect on World-level cooperation in our experiment (p = 0.043). In the second specification (see Table 2, column 2), we show that condition (2) holds as well, as IGI exerts a strong positive effect on GSI (p<0.001). Finally, the third specification confirms that condition (3) also holds (see Table 2, column 3). Once the GSI is introduced in the first model as a covariate, it exerts a strong effect on the dependent variable (p<0.001), while IGI loses its significance (p = 0.33). The three models studied control for a broad range of variables, namely, the global awareness index, NSI, LSI, demographic variables, a set of variables denoting an individual’s economic condition, and country dummies. We also included as covariate a measure of baseline cooperation at the local level drawn from the first experimental decision. That is the number of tokens contributed to the local account in a linear PGG. In this fashion, our dependent variable may be seen as measuring the propensity to cooperate at the global level that goes beyond the baseline propensity to cooperate at the local level. We preferred to deploy this measure of cooperation from the first decision as a control, rather than the measure of Local-level cooperation from the third decision, because the former measure is by construction independent from World-level cooperation. We further analyze Local-level cooperation in the third decision in the subsequent sections. All results are robust to omitting this measure of baseline local cooperation from the econometric models.

**Table 2:** Regression analysis of mediating effect of GSI between IGI and World-level cooperation.

DEPENDENT VARIABLE	Tokens contributed to world account	Global Social Identity	Tokens contributed to world account	Tokens contributed to world account	Tokens contributed to Personal account	Tokens contributed to Local account
	(1)	(2)	(3)	(4)	(5)	(6)
IGI	1.180**	0.417***	0.860	0.902	-1.272**	-0.614
	(0.547)	(0.0864)	(0.565)	(0.554)	(0.532)	(0.535)
GSI			0.993***	1.583***		
			(0.268)	(0.321)		
High Glob. Countries				0.999***		
				(0.268)		
GSI_X_High Glob. Countries				-1.181***		
				(0.437)		
Global Awareness Index	0.648**	0.274***	0.402	0.468	-0.233	-0.528*
	(0.294)	(0.0458)	(0.303)	(0.297)	(0.283)	(0.278)
NSI	0.412	0.465***	-0.0243	-0.106	-0.193	-0.0338
	(0.303)	(0.0488)	(0.337)	(0.337)	(0.270)	(0.327)
LSI	-0.207	0.0993**	-0.277	-0.304	-0.397	1.067***
	(0.272)	(0.0446)	(0.276)	(0.275)	(0.241)	(0.278)
Tokens contributed to Local account (First decision)	0.372***	0.0103***	0.362***	0.365***	-0.550***	0.0516*
	(0.0319)	(0.00340)	(0.0319)	(0.0320)	(0.0379)	(0.0281)
City	-0.350***	-0.0178	-0.353***	-0.371***	0.306**	-0.0355
	(0.131)	(0.0200)	(0.134)	(0.127)	(0.131)	(0.143)
Female	0.00554	0.0583***	-0.0489	-0.0569	-0.137	0.186
	(0.115)	(0.0179)	(0.117)	(0.114)	(0.109)	(0.121)
Education Medium	0.184	0.0222	0.172	0.0938	-0.104	0.157
	(0.151)	(0.0250)	(0.154)	(0.149)	(0.144)	(0.150)
Education High	0.221	0.0255	0.199	0.216	-0.0381	0.0166
	(0.152)	(0.0225)	(0.152)	(0.134)	(0.142)	(0.155)
Age Medium	-0.247*	-0.0230	-0.225	-0.233*	0.105	0.0616
	(0.144)	(0.0205)	(0.146)	(0.142)	(0.135)	(0.142)
Age High	-0.223	0.0409*	-0.244	-0.198	-0.0739	0.276*
	(0.154)	(0.0243)	(0.156)	(0.155)	(0.144)	(0.166)
Income Medium	0.109	-0.00566	0.100	0.0506	-0.0756	-0.167
	(0.142)	(0.0227)	(0.145)	(0.142)	(0.144)	(0.142)
Income High	0.0529	-0.0619**	0.100	-0.0190	-0.146	-0.186
	(0.194)	(0.0293)	(0.196)	(0.183)	(0.186)	(0.187)
Self Employed	0.130	0.0563*	0.113	0.0688	-0.0851	-0.104
	(0.213)	(0.0324)	(0.213)	(0.216)	(0.185)	(0.208)
Unemployed	0.527**	0.0380	0.470*	0.506*	-0.301	-0.268
	(0.248)	(0.0480)	(0.253)	(0.259)	(0.286)	(0.227)
Divorced	0.254	-0.0238	0.258	0.231	-0.147	-0.248
	(0.203)	(0.0276)	(0.203)	(0.198)	(0.198)	(0.205)
Russia	0.403*	0.121***	0.258		-0.355	-0.122
	(0.224)	(0.0309)	(0.231)		(0.217)	(0.218)
South Africa	0.129	0.0884**	0.0416		-0.172	0.496**
	(0.248)	(0.0403)	(0.253)		(0.237)	(0.222)
USA	0.673***	0.138***	0.539**		-0.721***	-0.411
	(0.245)	(0.0329)	(0.251)		(0.243)	(0.254)
Argentina	-0.215	-0.0166	-0.220		-0.271	0.633**
	(0.254)	(0.0381)	(0.255)		(0.232)	(0.253)
Italy	0.303	0.156***	0.138		-0.433*	-0.0789
	(0.229)	(0.0329)	(0.234)		(0.223)	(0.232)
Constant		-0.386***				
		(0.0548)				
Observations	983	978	976	976	983	983
Pseudo R^2^	0.0788	0.441	0.0815	0.0821	0.132	0.0207

**Note**: A tobit model has been used in the regression in column 2. The censoring values are the lowest and upper value for GSI, i.e. 0 and 1. An ordered logit model has been fitted to the regressions in other columns. Robust standard errors are in parentheses.

*** p<0.01,

** p<0.05,

* p<0.1.

The description of variables is in the Table E in S1 Appendix.

The Sobel-Goodmann test considers the difference in the coefficients for IGI in regressions (1) and (3) and checks whether the drop in the coefficient is large enough to be considered statistically significant. Other diagnostic variables check the validity of the overall model. The test strongly confirms that the coefficient difference is significant (p = 0.002; proportion of total effect that is mediated = 32%; bootstrapped std. err. with 1000 repetitions). This evidence supports the cosmopolitan hypothesis. Fig 1 plots the key relationships of the three econometric models that have been analyzed. In the panel (a) the effect of IGI on World-level cooperation in isolation from GSI is tested. Panel (b) illustrates both the *direct* and the *indirect* effect of IGI onto cooperation, once a GSI mediation effect is explicitly introduced in the analysis. It is worth noting that, in this case, while the indirect effect–i.e. the effect going from IGI to cooperation *through* GSI—is strongly significant, the residual effect–i.e. that going from IGI to World-level cooperation directly—is not statistically significant and thus, is fully mediated by the introduction of GSI in the model.

The fourth specification sheds more light on the nature of the relationship between GSI and propensity to cooperate globally (see Table 2, column 4). It introduces an interaction effect between the GSI and the three countries in our sample that have the highest level of globalization, as measured by the CGI–namely, the Russian Federation, Italy, and the US. This allows us to study whether GSI exerts differential effects in more highly-globalized countries vis-à-vis lesser-globalized countries. The answer is positive. GSI exerts a significantly stronger effect in countries at *lower* stages of globalization. This means that higher identification with the world as a whole has larger effects on World-level cooperation in countries that have a lower baseline level of globalization. For example, increasing one’s identification with the world community in Iran is associated with a propensity to cooperate globally that is significantly higher than increasing one’s identification with the world community in the US. We conclude:

**Result 3**: Our econometric and test analysis strongly supports the hypothesis that the GSI has a mediating effect between IGI and propensity to cooperate at the global level. This is consistent with the conjecture that participation in globalization increases propensity to cooperate at the global level as it simultaneously increases social identification with the world as a whole.
**Result 4**: GSI exerts larger effects in countries at lower stages of globalization than countries at higher stages of globalization.

### Robustness analysis: Test of mediation effects and total effects for alternative variables

Participation in global networks is itself a choice and so potentially endogenous to deeper preferences that may explain the association between IGI and World-level cooperation. A particularly important variable, in this respect, is one’s preference for participating and belonging to groups. This is related with what has been labelled ‘groupy behavior’ [51]. Similarly, differences in basic predisposition to generosity might explain the observed behavior. In other words, participating in global networks may be the consequence of deeper and more “hard-wired” personality traits that explains both the increased propensity to cooperate and the tendency to associate with global others.

Such personality traits—namely, the propensity to connect at the global level, the propensity to participate in groups, generosity, and finally the propensity to cooperate—are likely to be correlated with one another and it becomes difficult to single out which factor acts at a deeper level than others. Nevertheless, it is interesting to compare the relative strength of association of these constructs with World-level cooperation. In this section we will identify some proxies for each of the two possible additional explanatory variables, trying to “pit” them against IGI as factors explaining World-level cooperation.

Our post-experimental questionnaire included a set of questions asking whether the individual was a member of voluntary associations. Following a widely used taxonomy, we listed 13 different types of association and we asked subjects to state whether they were members of at least one association for each type (see Question 25 of questionnaire in S3 Appendix). We also asked subjects to state whether the type of association they joined were active at the local, national or global level (seeQuestion 26 of questionnaire in S3 Appendix). In the following analyses we use five variables as independent variables alternative to IGI. The first is a dummy variable that indicates whether an individual belonged to at least one association (“Association Membership”). The second is a dummy variable that indicates that an individual is a member of at least one association that is active globally, according to the participant’s opinion (“Global Association Membership”). The other two variables consider the number of *types* of associations to which an individual belonged to, divided by the number of possible types–i.e. 13. This scale has been often used as an index of the size of an individual’s social network, or social capital [52]. In our analysis we consider both “Number of Association Types” and “Number of Global Association Types”, that is, the number of types of associations a participant belonged to, and the number of types of global associations a participant belonged to, respectively. Table S5 reports a description and descriptive statistics for all these alternative variables.

To proxy for a subject’s generosity, we consider the answer to two questions asking whether the subject had contributed to either international aid efforts for natural disasters or for poverty relief (see Question 5a and 5b in S3 Appendix). We derive a variable “Donation Index” that is a summative scale of the dichotomous variables generated by answers to these questions. Table E in the S1 Appendix reports descriptive statistics for these variables.

Table 3 reports the results of our analysis. We perform three tests. First, we substitute one of these alternative variables for IGI in the mediation model. That is, an alternative variable enters as independent variable of the model while GSI keeps on being the mediating variable on “Cooperation” (see Table 3, columns 3, 5, 7, 9, 11). The second test adds IGI as a covariate to the previous model (see Table 3, columns 4, 6, 8, 10, 12). We can thus test the validity of a model having an alternative factor to IGI as independent variable while controlling for the effect of IGI. The third test considers both the original model where IGI acts as independent variable (Table 3, column 1), and the model where an alternative variable is added as covariate of the model (Table 3, column 2). We only report the results for adding “Association Membership” because this is the variable exerting the largest effects among the five additional variables being considered. We report the coefficient, standard error and P-values for the Sobel-Goodman mediation test, direct effect and total effect for each variable and for both models.

**Table 3:** Robustness analysis of mediation, direct and total effects for alternative independent variables.

	IGI		Association Membership		Number of Association Types		Global Association membership		Number of Global Association Types		Donation Index	
	(1)	(2)	(3)	(4)	(5)	(6)	(7)	(8)	(9)	(10)	(11)	(12)
	Without added control	With added control	Without added control	With added control	Without added control	With added control	Without added control	With added control	Without added control	With added control	Without added control	With added control
Sobel-Goodman test of mediation	0.074***	0.074***	0.074***	0.035**	0.074***	0.035**	0.039**	0.028**	0.074***	0.041**	0.074***	-0.049**
*Std*. *Err*.	*0*.*02*	*0*.*02*	*0*.*02*	*0*.*01*	*0*.*02*	*0*.*01*	*0*.*02*	*0*.*01*	*0*.*02*	*0*.*02*	*0*.*02*	*0*.*02*
P-value	0.003	0.002	0.004	0.015	0.07	0.015	0.015	0.037	0.003	0.013	0.005	0.010
Direct effect	0.16	0.15	0.11	0.094	-0.071	0.094	-0.032	-0.051	0.068	0.048	-0.067	-0.046
*Std*. *Err*.	*0*.*11*	*0*.*10*	*0*.*09*	*0*.*09*	*0*.*09*	*0*.*09*	*0*.*09*	*0*.*09*	*0*.*08*	*0*.*09*	*0*.*10*	*0*.*10*
P-value	0.13	0.14	0.22	0.28	0.43	0.28	0.70	0.57	0.42	0.58	0.51	0.65
Total effect	0.23**	0.22**	0.15*	0.13	-0.011	-0.040	0.008	-0.023	0.12	0.088	-0.13	-0.095
*Std*. *Err*.	*0*.*10*	*0*.*10*	*0*.*09*	*0*.*09*	*0*.*10*	*0*.*10*	*0*.*09*	*0*.*09*	*0*.*10*	*0*.*10*	*0*.*10*	*0*.*11*
P-value	0.022	0.035	0.086	0.14	0.91	0.68	0.93	0.79	0.20	0.36	0.22	0.37
Observations	976	976	976	976	976	976	976	976	976	976	973	973

**Note**: We report coefficients, standard errors and P-values for the Sobel-Goodman test of mediation, for the “direct effect”–namely, the effect of the independent variable after GSI is included in the model–and for the “total effect”—i.e. the effect when GSI is not included in the model. Standard errors for the Sobel-Goodman test and for the direct effect are bootstrapped with 1000 repetitions. The econometric model includes all the other covariates included in Table 2.

*** p<0.01,

** p<0.05,

* p<0.1.

The description of variables is in Table E of the S1 Appendix.

The main conclusion we can reach from this analysis is that the model with highest validity is the one with IGI as independent variable. Firstly, the total effect does not reach statistical levels of significance for any of the alternative variables being considered–except for “Association Membership” achieving weak levels of significance (p = 0.086)—when IGI is not included in the model. In contrast, the total effect for IGI is significant both without (p = 0.022) and controlling for “Association Membership” (p = 0.035). Finally, even if the Sobel-Goodman mediation test is statistically significant for all the variables considered, the significance is highest for the model with IGI as independent variable.

To have a sense of the relative effects of IGI and “Association Membership”, we consider the coefficients of their total effects. All variables have been standardized so their effects are directly comparable. An increase of one standard deviation of IGI increases propensity to contribute to the global account by 0.23 tokens (P = 0.022), while the increase of one standard deviation of “Association Membership” increases contributions to the global account by 0.15 tokens (P = 0.086). The effect of “Association Membership” is then about one third smaller than IGI. What is more, the two effects seem to be rather orthogonal to each other, as the total effect coefficients decrease only marginally once the alternative variable is included in the model. The total effect coefficient drops from 0.23 to 0.22 for IGI, and from 0.15 to 0.13 for “Association Membership”.

### Analyses of the impact of IGI on contribution to other accounts

In the above analysis our dependent variable was the contribution to the world account in the third decision of our experiment. Given the design of the decision, an increased contribution to global causes must be paid for by reductions in either the contribution to the local account or to the personal account. It is important to examine which one between these two accounts is affected by IGI. Individual globalization may be associated with a willingness to adjust contributions between public goods–so that more globalized individuals substitute contributions to the global account for contributions to the local account. Alternatively, individual globalization may go with increased generosity–so that more globalized individuals reduce their contributions to the personal account while keeping constant their contributions to the local public good.

Our econometric analysis supports the latter hypothesis. We replicate the same model used in Table 2, column 1, to estimate the effects of IGI on contributions to the world account, and we replace the dependent variable with contribution to the personal account (Table 2, column 5) and to the local account in Decision 3 (Table 2, column 6). We find that in both cases IGI exerts a negative effect, but this is statistically significant only for the personal account (p = 0.017), but not for the local account in Decision 3 (p = 0.25). Switching from a score of 0 in IGI to a score of 1 in IGI leads to a decrease of 0.31 (from 0.61 to 0.30) in the probability that an individual will give more than 3 tokens (the median of the distribution) to the Personal account. The same switch implies a drop in the probability of giving more than 3 tokens to the Local account of only 0.15 (from 0.62 to 0.47). Hence, to a large extent, increased IGI is associated with both increased contributions to the world account and reduced contribution to the personal account, leaving unaffected contributions to the local public good. This result helps characterize the psychological effects of participation in global networks. It suggests that greater involvement in global networks does not reduce propensity to cooperate with the local community. Rather, increased participation in global networks is associated with a genuine increase in overall generosity. This result further corroborates other findings on the positive association between identification with the whole humanity and generosity [10, 11].

## Discussion

The provision of public goods that are global in character calls for substantial cooperation at the global level by the relevant national parties [53]. Examples of such global public goods are the prevention of catastrophic climate change, a stable international financial architecture, averting the diffusion of contagious diseases, human security [54, 55]. Traditional policy analysis assigns national governments the duty to attain cooperation agreements to supply such public goods [56]. Nevertheless, in spite of global cooperation having developed in the last decades in many different arenas, ranging from climate change agreements to global vaccination programs, provision of global public goods today still falls very short, in the eyes of many, of the levels that are needed [57].

Partly as a result of the failure of national and intergovernmental action, direct action by individuals participating in formal or informal associations, or in self-coordinated forms of collective action, is seen as increasingly relevant for global cooperation. The actors behind these actions have been named “global civil society” [58–60]. The set of actors comprising global civil society is broad and includes environmental movements, labor unions, human rights promoters [61] and individuals active in so-called political consumerism [62,63].

In spite of the increased recognition of the role that individual citizens, alone or coalesced in movements or associations, play in global cooperation, our understanding of the mechanisms that shape the creation of such a global conscience, and the extent to which this generates public action, are little explored, let alone understood. The purpose of the present study has been to fill this gap using experimental methods.

More specifically, we have mapped the demographic characteristics, attitudinal values, and personality traits that are significantly correlated with our proposed index of global social identity and analyzed the linkages between participation in global networks, global social identity and propensity to cooperate at the global level. We found that individuals reporting high GSI scores expressed a positive view regarding global flows of people and objects and were more aware of global issues than individuals who have lower scores. Women and, although less robustly, older people and more highly-educated people tended to obtain higher GSI scores than others. We also found that GSI exerted larger effects in countries at lower stages of globalization than countries at higher stages of globalization. At the policy level, these results suggest that groups of countries who want to foster global cooperation may implement policies aiming to foster individual identification with the global community. According to Result 4, the benefits from these policies may be particularly high in countries with low levels of globalization. Admittedly, the engagement of individual citizens in global agreements for the provision of global public goods is currently rare [54]. Our findings provide evidence that higher engagement by the citizenry should on the contrary be sought after. In fact, identification with global communities is significantly lower than attachment to local and national communities in any country we surveyed.

As for the analysis of the cosmopolitan hypothesis, it had been demonstrated that (1) Increased participation in global networks is associated with increased propensity to cooperate at the global level. (2) Heightened identification with the global community is also associated with increased global-level cooperation [6,7]. In this contribution we have demonstrated a key relationship that corroborates the validity of the cosmopolitan hypothesis: (3) Global social identity exerts a mediating effect between participation in global networks and propensity to cooperate. This means that participation in global networks exerts an indirect effect on increased propensity to cooperate such that participation in global networks increases global social identity, which in turn increases propensity to cooperate globally. We hasten to say that the result of this test does not enable us to say that we have proved the existence of a causal relationship between the three variables at play. It will have the more modest, but arguably important, result of having ascertained that the evidence coming from our study is consistent with the cosmopolitan hypothesis, and therefore such a hypothesis has “survived” a relevant trial that may have led to its falsification.

The relevance of such a mediating mechanism also implies that increased participation in global networks is associated with the development of a sense of global social identity. This further undermines the ‘‘reactant” individual hypothesis, which posits increased entrenchment in local and national social identity as a result of globalization [6–8]. At least for those individuals who actively participate in globalization, higher levels of participation are associated with higher levels of cosmopolitan identity and global cooperation. We also demonstrated that such an increased propensity to cooperate at the global level does not come at the cost of reduced propensity to cooperate at the local level of interaction, but rather is associated with an overall increase in generosity. Finally, our robustness tests confirm the greater validity of IGI as an independent variable in comparison with other potentially relevant variables, such as the propensity to participate in associations and overall generosity.

Globalization is an all-encompassing process which is likely to affect fundamental aspects of human psychology. The results presented in this research confirm the existence of a strong and theoretically plausible association between participation in global networks, social identity, and propensity to cooperate with global others, paving the way for future research to gain better understanding of the underlying mechanisms.

## Supporting information

S1 Appendix Supporting tables. (PDF)

S2 Appendix Methodological notes on the construction of the CGI and IGI Indexes and on sampling strategy. (PDF)

S3 Appendix Research questionnaire. (PDF)

S4 Appendix Experiment script. (PDF)

S1 Data Dataset and codes to replicate econometric analysis. (ZIP)
