# Supplementary material for: Social identity mediates the positive effect of globalization on individual cooperation: Results from international experiments
Source: PLoS One. 2018 Dec 14;13(12):e0206819. doi: 10.1371/journal.pone.0206819 (PMC6294391; doi:10.1371/journal.pone.0206819)
Supplement: S1 Appendix — (PDF) [file pone.0206819.s001.pdf]

S1 Appendix

Supporting Tables

Table A: List of variables included in the Country-level Globalization Index (CGI)

Sub-Index	Variables
Economic globalization	Trade
	Foreign Direct Investment
	Portfolio Investment
	Non-residents income and income of residents working abroad
Social globalization (People)	Stock of Foreign Population
	Flow of Foreign Population
	Worker Remittances
	Tourists
Social globalization (Ideas)	Phone calls
	Internet users
	Films
	Books and newspapers
	Mail
Political globalization	Embassies
	UN Missions
	Organisations

Table B: Country scores for Country-Level Globalization Index

Country	Economic Globalization Score	Social Globalization Score	Political Globalization Score	CGI	Ordinal ranking
Singapore	*0.43*	*0.99*	*0.26*	*0.95*	*1*
USA	0.13	0.55	0.87	0.87	5
ITALY	0.13	0.40	0.71	0.67	13
RUSSIA	0.16	0.12	0.85	0.60	19
ARGENTINA	0.12	0.12	0.55	0.38	37
SOUTH AFRICA	0.14	0.07	0.51	0.34	48
IRAN	0.13	0.02	0.35	0.20	74
Samoa	*0.09*	*0.05*	*0.09*	*0.04*	*104*

Note: Table B reports scores for each sub-index of the Country-Level Globalization Index (CGI), the aggregate level of CGI and the ordinal ranking for the six countries included in our study and for the country at the top (Singapore) and at the bottom (Samoa) of the aggregate CGI at the time the research was run.

Table C: List of Questionnaire Items used to construct IGI

Description of items	Related question in questionnaire
Frequency of internet access	Question 1c
Territorial scope of phone use. Higher (lower) score if phone used at global level (is not used).	Question 2a
Territorial scope of mobile phone use. Higher (lower) score if mobile phone used at global level (is not used).	Question 2b
Territorial scope of email use. Higher (lower) score if email used at global level (is not used).	Question 2c
Territorial scope of mail use. Higher (lower) score if mail used at global level (is not used).	Question 2d
Territorial scope of fax use. Higher (lower) score if fax used at global level (is not used).	Question 2e
Speaking foreign languages	Question 15
Born abroad	Question 33a
Parent born abroad	Question 33b
Watch TV program from a different country	Question 6a
Read an international news source	Question 6c
Read international magazine	Question 6d
Read a book by foreign author	Question 6e
Listen to music made by foreign artists	Question 6f
Follow international news source	Question 6b
Travel abroad within continent	Question 3b
Travel abroad outside continent	Question 3c
Followed international sports events	Question 5c
Followed international cultural events or international trade fairs	Question 5d
Frequency with which participant goes to foreign cuisine restaurant	Question 11a/b A

Table C (continued)

Frequency with which participant has food/drinks made in a foreign country	Question 11a/b B
Frequency with which participant uses clothes made by foreign companies	Question 11a/b C
Frequency with which participant goes to restaurant owned by multi-national company (e.g. large fast food chains)	Question 12a/b A
Frequency with which participant uses food/drinks produced by multi-nationals	Question 12a/b B
Frequency of using clothes made by multi-nationals	Question 12a/b C
Work for multi-national company	Question 9
Car from other country	Question 10
Owns foreign currency	Question 14a
Owns bank deposit in other country	Question 14b
Owns investments in another country	Question 14c

Table D: Descriptive statistics of country samples, experimental decisions, and globalization indexes

Country/ Statistics	Obs.	Income [1=Highest Decile]	Education [1=Highest educational attainment]	Age	Gender [1=All females]	World-level Cooperation (Decision 3)	Local-level Cooperation (Decision 1)	IGI [1=Most globalized]	CGI [1=Most globalized]
IRAN	171								
Mean		0.31	0.43	38.36	0.50	3.49	4.85	0.29	0.20
St. Dev.		0.23	0.24	16.27	0.50	2.85	3.16	0.14	
Median		0.33	0.6	37		3	5	0.29	
SOUTH AFRICA	159								
Mean		0.58	0.35	36.98	0.65	3.81	5.58	0.41	0.34
St. Dev.		0.31	0.21	16.18	0.48	1.98	2.32	0.17	
Median		0.56	0.2	31		3	6	0.40	
ARGENTINA	201								
Mean		0.700	0.32	39.42	0.57	3.81	6.38	0.40	0.38
St. Dev.		0.27	0.21	11.98	0.50	2.84	2.61	0.13	
Median		0.78	0.2	39		3	6	0.41	

Table D (continued)									
Country/ Statistics	Obs.	Income [1=Highest Decile]	Education [1=Highest educational attainment]	Age	Gender [1=All females]	World-level Cooperation (Decision 3)	Local-level Cooperation (Decision 1)	IGI [1=Most globalized]	CGI [1=Most globalized]
RUSSIA	207								
Mean		0.57	0.44	40.71	0.57	4.70	6.47	0.39	0.60
St. Dev.		0.30	0.17	14.53	0.50	2.66	2.57	0.12	
Median		0.56	0.40	42		4	6	0.38	
ITALY	205								
Mean		0.50	0.42	40.39	0.52	4.49	6.07	0.42	0.67
St. Dev.		0.29	0.14	14.30	0.50	2.87	2.90	0.12	0.00
Median		0.44	0.40	40		4	6	0.419	
USA	171								
Mean		0.36	0.40	40.59	0.47	5.80	7.49	0.41	0.87
St. Dev.		0.25	0.24	15.71	0.50	3.16	2.53	0.11	
Median		0.33	0.4	40		5	8	0.39	

Table D (continued)									
Country/ Statistics	Obs.	Income [1=Highest Decile]	Education [1=Highest educational attainment]	Age	Gender [1=All females]	World-level Cooperation (Decision 3)	Local-level Cooperation (Decision 1)	IGI [1=Most globalized]	CGI [1=Most globalized]
All countries	1114								
Mean		0.51	0.40	39.53	0.54	4.35	6.16	0.39	0.52
St. Dev.		0.31	0.21	14.80	0.50	2.86	2.80	0.14	0.22
Median		0.44	0.40	39		4	6	0.383	

Note: Obs. is the number of observations and is taken from variable 'World-level Cooperation'. Missing variables may occur for other variables. All variables apart from 'Age', and 'World-level Cooperation' and 'Local-level Cooperation' are scaled on the [0 , 1] interval. 'Income' denotes the income decile to which a participant responded s/he belongs within his/her country income distribution (see Question 38 in the research questionnaire reported in the S3 Appendix). 'Education' is the highest level of education attained by an individual (see Question 32 in research questionnaire in the S3 Appendix). 'Age' is the participant's age (see Question 31 in research questionnaire in the S3 Appendix). 'Gender' is a dummy variable identifying with the value of 1 (0) females (males). 'World-Level Cooperation' and 'Local-Level Cooperation' is the number of tokens contributed to the World (Local) Account out of 10 available tokens in the third (first) decision of the experiment (see S4 Appendix). 'IGI' is the score for the Individual-Level Globalization Index (See Table C). 'CGI' is the score for the Country-level Globalization Index (Lockwood and Redoano, 2005; see Table A). The whole research questionnaire is reported in the S3 Appendix.

References

Lockwood, B. and M. Redoano, 2005 (http://www2.warwick.ac.uk/fac/soc/csgr/index/).

Table E: Description and descriptive statistics of variables included in the econometric analysis

Name of variable	Description	Mean value	Median value	Std. Dev	Minimum value	Maximum value	N. obs.
Age High	Dummy variable, where 1 identifies people older than 50.	0.26		0.44	0	1	1092
Age Medium	Dummy variable, where 1 identifies people between 31 and 50 years old.	0.40		0 .49	0	1	1092
Association Membership	Dummy variable identifying whether the participant belongs to at least one of the association types listed in Question 25a-m of Questionnaire.	0.68	1	0.47	0	1	1122
CGI	Country-level Globalization Index. It offers a ranking of countries on a [0, 1] interval according to their level of economic, social and political international interconnectedness (see Lockwood and Redoano, 2005).	0.52	0.60	0.22	0.20	0.87	1122
City	Dummy identifying people living in the largest urban center sampled in each country	0.65		0.48	0	1	1122
Divorced	Dummy identifying people who are divorced	0.097		0.30	0	1	1095
Donation Index	Number of "Yes" answers to questions on whether participant contributed to international aid efforts for natural disasters or poverty reliefs. See Questionnaire: Questions 5a-b.	1.27	1	0.78	0	2	1087
Education High	Dummy variable, where 1 identifies bachelor or higher degree.	0.36		0.48	0	1	1102
Education Medium	Dummy variable, where 1 identifies upper secondary school degree.	0.26		0.44	0	1	1102
Entry	Participant agrees with the following statement: "We should restrict and control entry of people into our own country more than we do." See Questionnaire: Question 27c.	2.79	3	1.065	1	4	1093
Female	Dummy variable, where 1 (0) identifies female (male) participants.	0.54		0.50	0	1	1098

Table E (continued)

Name of variable	Description	Mean value	Median value	Std. Dev	Minimum value	Maximum value	N. obs.
Foreign Immigrants	Dummy variable assigning value of 1 if participant reports that some groups of foreign migrants live in the area where the participant lives. See Questionnaire: Question 16a.	0.82		0.38	0	1	1085
Global Association Membership	Dummy identifying whether at least one of the association types that the participant belongs to – as per Question 25a-m-, is active internationally, as per Question 26.	0.23	0	0.42	0	1	1122
Global Awareness Index	The index measures the level of participant's awareness with the following global issues: global warming, the spread across the planet of potentially dangerous diseases, the action of the International Criminal Courts of justice, the persistent gap between rich and poor people around the world. The index assigns value of 1 if the participant does *not* answer that "she is not informed about this issue" to the four items of question 4 of the questionnaire. It then sums up the four resulting scores. Therefore, the index is equal to 0 (1) if the participant is not informed on any of these issues (is informed of all these issues). See Questionnaire: Question 4.	0.70	0.75	0.21	0	1	1100
Global Social Identity (GSI)	Index of Global Social Identity. See sections 2.2 and 3.2.	0.54	0.56	0.28	0	1	1092
Global Social Identity_X_High Glob. Countries	Interaction variable between Global Social Identity and High_Glob_countries.						1092
High Glob. countries	Identifies the three most globalized countries in our sample, i.e. the US, Italy, and Russia						1122
Income High	Dummy variable, where 1 identifies income levels higher than the seventh decile of a country's income distribution. See Questionnaire: Question 38.	0.28		0.20	0	1	1057

Table E (continued)

Name of variable	Description	Mean value	Median value	Std. Dev	Minimum value	Maximum value	N. obs.
Income Medium	Dummy variable, where 1 identifies income levels between fourth and seventh deciles of a country's income distribution. See Questionnaire: Question 38.	0 .44		0 .50	0	1	1057
Individual Globalization Index (IGI)	See section 3.2.	0.38	0.38	0.14	0.011	0.83	1115
Local Social Identity (LSI)	Index of Local Social Identity. See sections 2.2 and 3.2.	0.70	0.67	0.27	0	1	1107
National Social Identity (NSI)	Index of National Social Identity. See sections 2.2 and 3.2.	0.72	0.78	0.24	0	1	1097
Number of Association Types	Counts the types of association the participant belongs to in Question 25a-m of Questionnaire.	0.16	0.09	0.18	0	1	1122
Number of Global Association Types	Counts the types of association the participant belongs to in Question 25a-m of Questionnaire that are active internationally, as per Question 26.	0.03	0	0.08	0	1	1122
Opinion Glob.	Participant agrees that the following two statements are a "very good thing": "The world becoming more connected through greater economic trade and business ties."; "The world becoming more connected through faster communication and greater movements of people". Answers were given on a 4-point Likert scale ranging from "Very Bad" to "Very good". Scores in these two items have been summed. See Questionnaire: question 28.	2.08	2	1.21	1	5	1096
Self Employed	Dummy identifying people who are self-employed. See Questionnaire: Question 36, 37.	0.11		0.32	0	1	1071
Unemployed	Dummy identifying people who are unemployed. See Questionnaire: Question 36.	0.053		0.22	0	1	1071
Way of Life	Participant agrees with statement "Our way of life needs to	2.51	3	1.066	1	4	1094

	be protected against foreign influence.” See: Questionnaire: Question 27b.						

Note: Variables are listed in alphabetical order.

Table F: Results of statistical tests regarding distribution of social identity measures

Panel a: Iran (N=176)

	Local Social Identity	National Social Identity
National Social Identity	-1.45<br>(0.15)	-
Global Social Identity	9.32<br>(<0.0001)	10.52<br>(<0.0001)

Panel b: South Africa (N=142)

	Local Social Identity	National Social Identity
National Social Identity	-0.35<br>(0.7282)	-
Global Social Identity	4.74<br>(<0.0001)	5.66<br>(<0.0001)

Panel c: Argentina (N=196)

	Local Social Identity	National Social Identity
National Social Identity	-0.44<br>(0.6635)	-
Global Social Identity	9.48<br>(<0.0001)	11.13<br>(<0.0001)

Panel d: Russian Federation (N=205)

	Local Social Identity	National Social Identity
National Social Identity	3.63<br>(0.0003)	-
Global Social Identity	7.89<br>(<0.0001)	7.56<br>(<0.0001)

Panel e: Italy (N=203)

	Local Social Identity	National Social Identity
National Social Identity	-3.54<br>(0.0004)	-
Global Social Identity	4.27<br>(<0.0001)	6.83<br>(<0.0001)

Panel f: US (N=171)

	Local Social Identity	National Social Identity
National Social Identity	-0.65<br>(0.51)	-
Global Social Identity	1.82<br>(0.070)	3.01<br>(0.0026)

Panel g: All (N=1086)

	Local Social Identity	National Social Identity
National Social Identity	-1.29<br>(0.20)	-
Global Social Identity	15.64<br>(<0.0001)	18.94<br>(<0.0001)

Note: The table reports z-values and p-values (in brackets) of non-parametric Wilcoxon signed-ranks test tests over matched pairs of observations. The null hypothesis is that observations for the two variables object of the test have the same distribution. For instance, the values at the intersection of "Local Social Identity" (column entry) and "National Social Identity" (row entry) report the test on the null hypothesis that "Local Social Identity" and "National Social Identity" have the same distribution for a specific country. A positive (negative) value implies that observations for the variable in the column entry tend to be higher (lower) than the observations for the variable in the row entry. The number of observations for each country is reported close to the title of each panel.
