# Supplementary material for: Social identity mediates the positive effect of globalization on individual cooperation: Results from international experiments
Source: PLoS One. 2018 Dec 14;13(12):e0206819. doi: 10.1371/journal.pone.0206819 (PMC6294391; doi:10.1371/journal.pone.0206819)
Supplement: S2 Appendix — (PDF) [file pone.0206819.s002.pdf]

S2 Appendix

Methodological notes on the construction of the CGI and IGI Indexes and on sampling strategy

The Country-level Globalization Index (CGI)

The Country-level Globalization Index (CGI) developed by the Centre for the Study of Globalisation and Regionalisation (CSGR) (Lockwood and Redoano, 2005) gauges the economic, social and political dimensions of globalization for a sample of 104 countries and combines them into an overall globalization index for each country for the years 1982-2004. The list of variables on which the index is computed is reported in Table A in the S1 Appendix. Scores for each of the economic, social, and political domains are computed for each country. These are then combined to yield the overall Country-Level Globalization Index (CGI). Such scores are reported in Table B in the S1 Appendix, along with the ordinal ranking of a country within the sample. For comparative purposes, we have also included the scores for the country at the top (Singapore) and at the bottom (Samoa) of the overall ranking. Our sample of countries, though numerically limited, covers a broad range of the globalization spectrum - although admittedly countries at the bottom end of the scale are under-represented.

The Individual-level Globalization Index (IGI)

The Individual-level globalization index (IGI) was constructed to be analogous to both the CSGR (Lockwood and Redoano, 2005) and the Foreign Policy (Kerney, 2006) indexes of country-level globalization, although some importance differences between the individual and country-level constructs remain. The content of the questionnaire is specified by the theoretical conceptualization of globalization outlined in the paper. The IGI measures the extent to which individuals interact with and potentially become interdependent with distal others in economic, social and cultural interactions. The 30 items forming the IGI are reported in S3 Appendix.

Most questions on the IGI are Likert scaled, with the lowest category denoting a lack of ownership or access of a particular medium of connection and highest category denoting the highest frequency of use or interaction (see e.g. Question 3b in questionnaire in the S3 Appendix). Some questions inquire as to the scope of activity; for example, whether someone uses their mobile phone to contact people in their locality, other parts of their country, or other countries (see e.g. Question 2b in questionnaire in the S3 Appendix). Finally, other questions are “yes” / “no” in response format; for example, whether someone works for a multinational company; these questions purely gauge participation or non-participation in globalization (see e.g. Question 9 in questionnaire in the S3 Appendix).

The scores to each question have been reverse-scored when necessary, and normalized to the [0,1] interval, such that a score of 0 always corresponds to the lowest possible occurrence of an event or circumstance - e.g. a lack of access to an international news source, and a score of 1 is associated with the maximum possible occurrence - e.g. highest possible frequency in watching

or listening to an international news source. The normalized scores have then been summed up and divided by the number of the questions answered by the individual. As for the multiple choice questions asking the area (local, national, international) in which a subject carries out a certain activity, we assigned a lexicographic score reflecting the broadest area within which the subject has interactions. That is, scores of 1/0.5/0.25 were assigned if the subject answered she has international/national/local interactions, respectively, and a score of 0 if the subject has no such interaction.

For further details on the index construction, see the notes at the end of the questionnaire in the S3 Appendix.

Further details on sampling strategy

As illustrated in the paper, we used a random quota sampling method to recruit participants. The three chosen criteria (gender, age, and socio-economic statues) yielded a grid with 18 cells to be filled with equal numbers of participants in each cell, to the maximum extent possible. The manner of ascertaining the SES of participants was left to the local researcher, who determined which method or question was most culturally appropriate. Most often this question concerned education or type of employment (serving as proxies for low or high Socio-Economic Status), and income. In Argentina, Italy, and Russia, recruitment was carried out by agencies specialized in survey polls and market research. Sampling generally happened in two stages: in the first contact, the position of a person who was available to participate in the research was ascertained with respect to the three criteria above. Had the ‘cell’ in the grid occupied by the participant been filled already, then the participant would have been turned down. Survey agencies already had assignments of participants to SES category from previous evaluations. All participants were screened to have at least a fourth-grade education and had lived in their locality of residence for at least one year at the time the research was conducted.

An experimental protocol, which explains how to conduct the various phases of the research, was distributed to local researchers in each country. This is reported in the S4 Appendix.

References

Kearney, A. T., (2006). Globalization index. Foreign policy, 157, 74-81

Lockwood, B. and M. Redoano, 2005 (http://www2.warwick.ac.uk/fac/soc/csgr/index/).
