# Supplementary material for: Social identity mediates the positive effect of globalization on individual cooperation: Results from international experiments
Source: PLoS One. 2018 Dec 14;13(12):e0206819. doi: 10.1371/journal.pone.0206819 (PMC6294391; doi:10.1371/journal.pone.0206819)
Supplement: S4 Appendix — (PDF) [file pone.0206819.s004.pdf]

S4 Appendix

Experiment Script

Summary of Experiment Protocol

The research was conducted between May and September 2006. Local researchers were native to the place where the research was conducted. Experiment control was guaranteed by the presence of a member of the core research team during all experimental sessions in each location.

An experiment session lasted around an hour, and comprised three experimental decisions and the completion of the questionnaire. Participants were paid the purchasing power equivalent of US$8.00 as a show-up fee as they entered the experiment room. Participants randomly chose an identification number to identify themselves throughout the experiment; never were participants' names or other personally identifying information provided to the researchers. Furthermore, to guard against any possible political risk to participants in Iran and Russia, recruitment lists were destroyed at the beginning of each experimental session in view of participants.

To the extent possible, subjects were isolated from one another so that privacy was maintained throughout the experiment. Instructions for the tasks were delivered orally by a native speaker of the language in which the experiment was conducted. A written comprehension check consisting of three questions regarding the basic logic and procedure of the Multi-level Sequential Cooperation (hereafter MSC) game was administered to subjects after providing instructions for the decision. This was collected and saved by the experimenter so that decisions of subjects who had failed the test could be expunged from the dataset at the end of the session. Shorter comprehension checks were also conducted after the instructions for second and third decisions to make certain subjects understood the basic logic of the MSC game. The correct answers to the questions were communicated before subjects made their choices.

Subjects made three experimental decisions in a fixed order. Pilot tests found no ordering effects. Decisions were anonymous, and the groups to which subjects were assigned were randomly selected at the beginning of each decision. No feedback between decisions was provided. Hence, the three decisions can be treated as independent. The first decision measured propensity to cooperate with people living in the same locality through a non-nested MSC. The next two decisions examined how much individuals were willing to cooperate beyond their locality with people coming from other areas of their nation and other parts of the world. We used an MSC experiment at the national and global level for this purpose with identical monetary incentives.

Decisions were made privately using tokens that could be allocated into envelopes representing the personal, local, national and global accounts. In Iran, due to some logistical impediments, subjects made their choices with pen and paper. After subjects completed the three decisions there was a waiting period while their outcomes were determined. It was during this

waiting period that subjects completed the questionnaire from which the IGI and additional demographic information were derived. When necessary, the questionnaire was read aloud as participants followed along and made their responses. Average take-home earnings from the experiment were the purchasing power equivalent of US$34.00.

Research Participant Information and Consent Form

<Note: The information and consent form reported in the following page was distributed in the US location of Madison, Wisconsin. The parts highlighted in yellow were replaced with relevant information pertaining to other locations.>

UNIVERSITY OF SOUTH CAROLINA
Research Participant Information and Consent Form

Title of the Study: A Worldwide Exploratory Inquiry into the Influence of Globalization on Decision Making

Principal Investigator: Nancy R. Buchan (phone: 803-777-1781) (email: nancy.buchan@moore.sc.edu)

DESCRIPTION OF THE RESEARCH

You are invited to participate in a research study about how people around the world make decisions. Specifically your decisions will involve people from this local community, from this country and from other countries. You and the others participating in this experiment can make money depending on the decisions each of you make.

You have been asked to participate because we are interested in studying the attitudes and behaviors of people from different parts of the United States, and in different regions within a number of other countries around the world.

This study will include adults of all ages, economic classes, and races from within a number of countries around the world.

The research involves two parts, an experiment, and a questionnaire. The experiment will take place in the meeting room you are about to enter, if you consent to participate in the experiment. After completing the experiment you will be asked to fill out a questionnaire.

WHAT WILL MY PARTICIPATION INVOLVE?

If you decide to participate in this research you will be asked to make a series of decisions regarding how to allocate money (given to you by the experimenter). This money will be shared locally, nationally, or with people from around the world. You will also be asked to complete a questionnaire regarding your participation in various activities (for example, the number of times a month you communicate with someone in another country), and regarding your attitudes toward some current issues.

Your participation will last approximately 90 minutes in total.

ARE THERE ANY RISKS TO ME?

There are no risks involved in participation in this research. You will be provided money to allocate in the experiment, and thus, you will not use your own money. All money that you earn in the experiment is yours to keep. Additionally, any behavior or responses during this experiment will be kept completely confidential and anonymous. You will be identified in the research only by a experiment ID number, and there will be no record linking your actual identity with your responses.

ARE THERE ANY BENEFITS TO ME?

You are free to take home any money you receive during this experiment.

You will receive a show up fee plus any money received during the experiment for participating in this study.

If you do withdraw prior to the end of the study, you will receive no compensation beyond the show up fee.

HOW WILL MY CONFIDENTIALITY BE PROTECTED?

While there will probably be publications as a result of this study, your name will not be used. Only group characteristics will be published.

WHOM SHOULD I CONTACT IF I HAVE QUESTIONS?

You may ask any questions about the research at any time. You will now be given a copy of this form along with the business card of the local researcher connected with this project which contains the following information:

Nancy R. Buchan, Associate Professor
Sonoco International Business Department Moore School of Business University
of South Carolina
1705 College Street
Columbia, SC 29208
Tel: 803-777-1781
Fax: 803-777-3609

If you have any questions about the research after you leave today you should contact him/her.

Your participation is completely voluntary. If you decide not to participate or to withdraw from the study there will be no adverse consequences.

If you consent to participation in this research, you may walk into the experiment room now. Doing so indicates that you have read this consent form, had an opportunity to ask any questions about your participation in this research and voluntarily consent to participate.

If you do not consent to participate in this research, you are free to leave now. We thank you for your time and interest.

Experiment script (Version used in a US location)

<Note: Instructions to experimenters are in italics. Text in non-italics is read to the Ss.>

As Ss arrive, welcome them and hand them a consent form. If they cannot read the Consent form, then have it read to them. Typically this form will be read outside the experiment room. The Consent form notes that if they enter the room, they are agreeing to participate; Ss will not sign the form, only non-verbal consent is necessary. Local custom will dictate what should be done.

If Ss refuse to participate, then pay the show-up fee and send them on their way.

Once Ss are seated, pay them their show-up fee. Once paying each, have the Ss randomly draw a sheet of stickers with their ID #. (The stickers may be placed down on a table to allow Ss to randomly draw them).

First please take your show up fee out of the envelope and put it away, this money is yours to keep for coming to the experiment. Next, please draw a sheet of stickers. These stickers will have your ID number on them. You will notice you have 12 stickers on the sheet. These will be used later for your decisions in the experiment; you will put the ID stickers on different pieces of paper and on envelopes.

Now please place one ID sticker on the empty envelope. We will collect these envelopes now and at the end of the experiment, each envelope will be returned – containing the experiment earnings - to the person with the matching ID number. We will not know who you are or what decisions you made – we will only know your ID number.

The experimenter should collect the envelopes.

Please turn your sheet over and do not show it to the others. Do not tell anyone, except the experimenter, your number. Please wait until everyone has arrived and then we can get started. Please keep quiet and do not speak to the others in this room.

When everyone has arrived and is seated then the instructions can begin.

Welcome to this research project. An international team of researchers is looking at the way in which people in this <local community>, this <COUNTRY NAME> and around the world make decisions. If you pay close attention to the instructions then you could make a significant amount of money.

The research team that is here today includes myself <give name>, an assistant <give name> and <give name of Core member> along with another assistant <give name> who will be outside the room making your payments.

Everyone should be present in the room at this point and acknowledge the introduction. The Core member and the second assistant should then leave.

In this project you are going to be asked to make decisions with other people. Some will be in this local community, but they may not be in this room now; some will be from this <COUNTRY NAME> and some will be from countries around the world. Many people have already made their decisions and other groups are doing the same research this week. Your choices, and the choices by others, will be matched with the help of a colleague at another university when you are finished. You will be paid in cash at the end of this research for the decisions that you and the people you have been matched with made.

The same instructions are being given to other people in other countries. That is why we are reading this script. Everyone is hearing the same thing you are, except in their own language.

All of the decisions are similar, so please pay attention to these instructions. At the outset of each decision you will be given 10 colored tokens. Everyone will get the same materials that you get. It will be important to keep in mind that colored tokens are worth <$.50> each to you. For other people, whether from around here, in the region or around the world, their colored tokens also are worth money to them. We have taken care that their tokens, once converted to their foreign currency, are worth the same value as your tokens in terms of what could be purchased with them. That is, people in other countries will receive an amount in their currency such that they can buy in their country the same amount of goods that <*$.50*> will buy in the United States.

Again, keep in mind that you are being matched with other people (some of whom are from around here and some of whom are from around the world). What those people have decided to

do and what you will decide to do affects how much you can make. When your decisions are submitted, our core team member will be using our computer connection to receive information about others' choices in order to calculate each person's payments. This may take a little while so please be prepared to wait for a few minutes at the end of the session so that we can give you your final payment before you leave today.

After a pause, begin the instructions…

Your task is to decide how you want to allocate your tokens between different envelopes. You will have several options, sometimes two and sometimes three. Here I will explain the simplest decision where there are only two ways to allocate your tokens.

DECISION ONE.

In first decision you will be given 10 tokens, and you can put your tokens into your "Personal" envelope or into your <Local> envelope. The number of tokens you put into any envelope is entirely up to you.

What's the difference between the envelopes? Whatever you put into the "Personal" envelope is yours and will not be shared with anyone else. As mentioned before, for every colored token you put into that envelope is worth <$.50> to you regardless of the other people's decisions. Now, what about the <Local> envelope? Any colored tokens that you and three other people put into your <Local> envelopes will be doubled by me. You and the other three people will get an equal share of that amount.

Where do these three other people come from? As I mentioned, you are going to make this decision with 3 other people. They may not be in this room, but they are from this local area. I do not know which people you will make decisions with because you will be mixed with lots of other people in order to make a group of four. All of the people you are mixed with are from around here.

At this point the local helper should begin passing out the materials (including the example sheet for Decision 1). This material should all be bundled together, except for the comprehension sheet, to make it easy to pass out.

I am now going to pass out your materials. You should have an envelope marked "Personal" and an envelope marked *<Local>*. You should have 10 red tokens (each of which are worth <$.50> to you and everyone else). Your tokens are in your "Personal" envelope. Please take them out and count them to make certain you have 10.

The first thing I would like you to do is take two stickers off your ID card and put it on the upper right corner of both your envelopes. Please make certain you do this. This is the only way we can make certain you will be paid.

At this point the experimenter can demonstrate how this is done on a blank envelope.

Also, it is important that you do not write on, fold, or damage the envelopes in any way. Only your ID sticker should be on the envelope.

Before you make your decision, I want to make certain you understand how you get paid. Please make certain you know exactly how you can receive money. You will be paid based on the decisions that you and the others you are mixed with make.

Once I am finished with the examples you will make your own decision about how many red tokens you will put in your "Personal" envelope and how many tokens you will put in your <*Local*> envelope.

Please follow along with the examples that have been handed out to you. For example #1, suppose that you put 10 red tokens in your "Personal" envelope and the other three people put a total of 12 red tokens in their <Local> envelopes. In that case, the 12 tokens in the local pot will be doubled (to 24) and shared equally among you and the other three people (6 each).

Example 1

You put 10 red tokens in your "Personal" envelope. Others put a total of 12 red tokens in their <Local> envelopes. Those <Local> envelope tokens are doubled and you get an equal share.

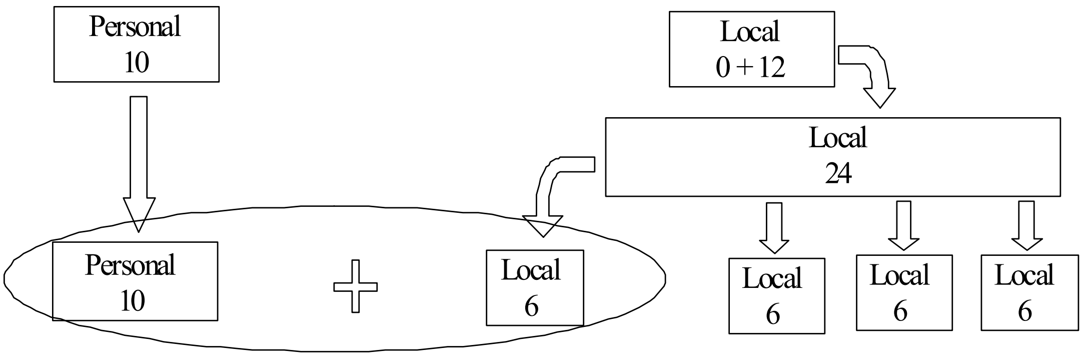

You would then receive a total of 16 tokens: 10 from your personal envelope that you kept, and 6 from your share of the local pot; given that there were 12 red tokens in the <Local> envelopes, that amount would be doubled to 24 by me and you would get an equal share, which is 6 red

tokens. You would end up with 16 tokens worth <$8.00>. Is anyone uncertain about how this happens?

To take another simple example (#2) suppose you put 8 of your red tokens in the <Local> envelope and no one else put any red tokens in the <Local> envelope. What would you receive? If you like you can write in the blanks on the example.

Example 2

You put 2 red tokens in your "Personal" envelope. Others put 0 red tokens in their <Local> envelopes.

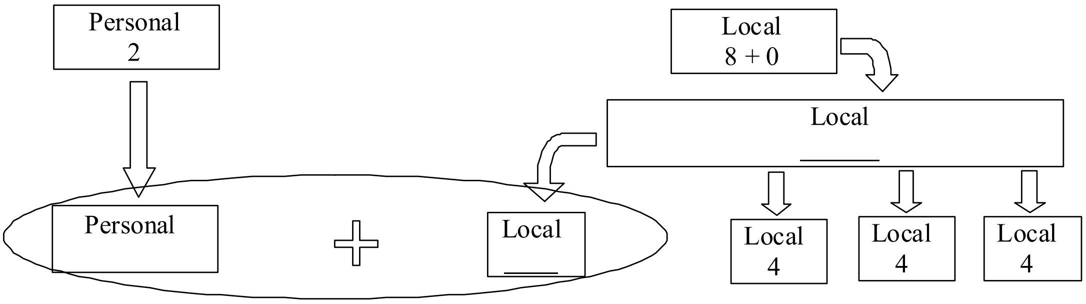

Wait while participants make calculations; look around to see if they are attempting to come up with the answer; encourage someone to give an answer.

You would receive a total of 6 tokens. First you would have 2 red tokens in your "Personal" envelope. Given that there were 8 red tokens in the <Local> envelopes (all put there by you) that amount would be doubled to 16 and you would get an equal share, which is 4 red tokens. The other people in your local group also get 4 red tokens. You would end up with 6 tokens worth <$3.00>.

Finally, let me give one more example (#3). Suppose you put all 10 of your red tokens in the <Local> envelope. Suppose that the other 3 people did the same thing. That means a total of 40 red tokens in the <Local> envelopes. How much would you receive?

Example 3

You put 0 red tokens in your "Personal" envelope. Others put 30 red tokens in their <Local> envelopes.

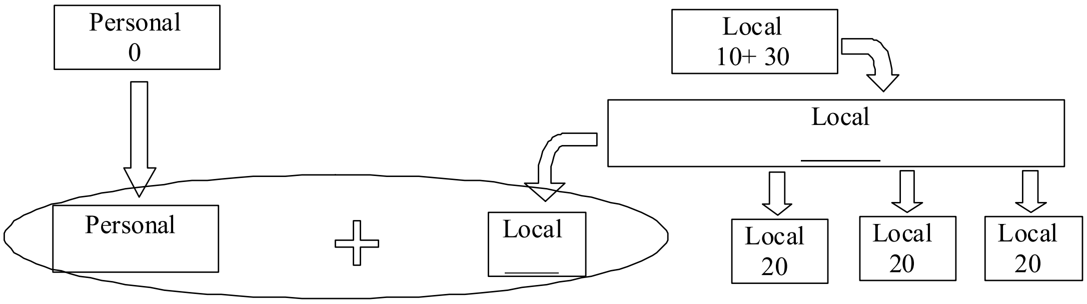

Wait for an answer from participants…

You (and the other three people) would receive a total of 20 tokens, which is <$10.00> for this decision. First you would have 0 red tokens in your "Personal" envelope. Second, in the <Local> envelopes there would be 40 tokens. This would be doubled to 80 and your share would be 20 tokens.

These three examples show that what you get can be very different, depending both on what you and everyone else does. Please take the time to look through the examples. Do this while we are passing out a new sheet of paper.

At this point pass out the comprehension sheet.

A sheet is being handed out to you with three questions on it. Please do not answer the questions until I read them aloud.

When the sheets are handed out, begin the instructions.

Before you do anything, please remove one of your stickers and put it in the upper right hand corner of the sheet that was just handed out.

Pause until everyone has done so.

I am going to read the questions one at a time. Please check the answer you think is most appropriate. When everyone is done I will read the answers.

1. What happens when a red token is put into the <Local> envelope? (Nothing; The token is cut in half; The token is doubled; The token is tripled)

2. How many people, including you, are in the <Local> group? (Two people; Three people; Four people; Five people)

3. Everyone gets an equal share of the <Local> envelope. (True or False?)

Pause until everyone is finished. These sheets will be collected with the envelopes.

Now that you are done, I will read the answers. Do not mark your papers. They will be collected later.

1. What happens when a red token is put into the <Local> envelope? The red token is doubled and your get an equal share along with the other people in the <Local> group.

2. How many people, including you, are in the <Local> group? There are a total of four people in the local group. This includes you. This means each of you will get a one-quarter share of the red tokens that are put into the local envelopes and doubled.

3. Everyone gets an equal share of the <Local> envelope. This is true – everyone gets an equal share.

Now it is time for you to make your decision. You can put any combination of tokens into the 2 envelopes. Remember that the red tokens you put into your "Personal" envelope are yours and will not be divided among any others. Whatever you and the three other people from around this area put into the <Local> envelopes will be doubled. Each of you will get an equal share of that amount. Please make your decision and then place the envelopes on your [box/desk]. DO NOT seal the envelopes. My assistant will come around and collect your envelopes and all your materials. The assistant will check to make certain you have put your ID number in the upper right corner of your envelopes. When you have finished put your envelopes on top of your [box/desk] so we will know you are finished. If you have any questions please raise your hand.

> *The envelopes will be put into a box marked Decision 1. The assistant should double check each envelope to make certain that it has an ID number attached to it. The comprehension sheet should also have an ID on it. If not, ask the subject to do it before the envelopes are placed in the box. Also collect any other materials from the subjects.*
>
> *The envelopes should be taken to a Core member of the team who is outside the room. The Core member should open the personal envelopes and enter and record the number of red tokens for each subject. The Core member should then open the <local> envelope, enter and record the number of red tokens, check the group assignment and calculate the share obtained by the S. The personal tokens and the Ss group share should be filled out on the decision record slip of paper for each participant.*
>
> *The data for each participant's choices and payment should also be entered onto the session spreadsheet.*

Now that everyone's decision has been made, the envelopes will be matched with other people and how much money you receive will be calculated. It will take a while to do this. At the end of the session you will be given an envelope with your payment.

DECISION TWO.

You have now finished the first decision. The second decision is slightly different, so please listen very carefully. In this decision you will have 10 blue tokens and 3 envelopes. Once again you will be paid <$.50> for each blue (colored) token.

In this decision you will be randomly mixed with different groups of people. The first group will be similar to the first decision. You will be mixed with three other people from this local area. It is very likely that this will be three different people than the first time. The second group will be composed of 12 people. It will include the three local people, plus two other groups of four people from other areas in this country.

As with the first decision, the blue tokens you put in your "Personal" envelope will be yours and not divided with anyone else. Second the blue tokens you and the others put into the <Local> envelope will be doubled and you will get a 1/4 share from the local group. Finally you have a <COUNTRY NAME> envelope. The blue tokens that all 12 people put into those envelopes will be tripled. You will get an equal share of the tripled amount.

The assistant hands out the bundle of materials (including the example sheet for Decision 2); Ss each get a bundle.

Your task is to put 10 tokens in the envelopes. You can put them in any combination that you please. My assistant will now hand out these materials. You should get 10 blue tokens and 3 envelopes. You should have 10 blue tokens (each of which are worth <$.50> to you and everyone else). Your tokens are in your "Personal" envelope. Please take them out and count them to make certain you have 10.

Please remove an ID sticker and put one on each of the three envelopes. Please do this now and make certain it is in the upper right corner.

Please follow along with the examples that have been handed out to you. For example 1 suppose you put 10 blue tokens in your "Personal" envelope, others put 10 blue tokens into their <Local> envelopes and 12 blue tokens were put into the <COUNTRY NAME> envelopes. How much would you receive?

Example 1

You put 10 blue tokens in your "Personal" envelope. Others put a total of 10 blue tokens in their <Local> envelopes. Finally, 12 blue tokens are put in the "US" envelopes. Those "US" envelope tokens are tripled and you get an equal share.

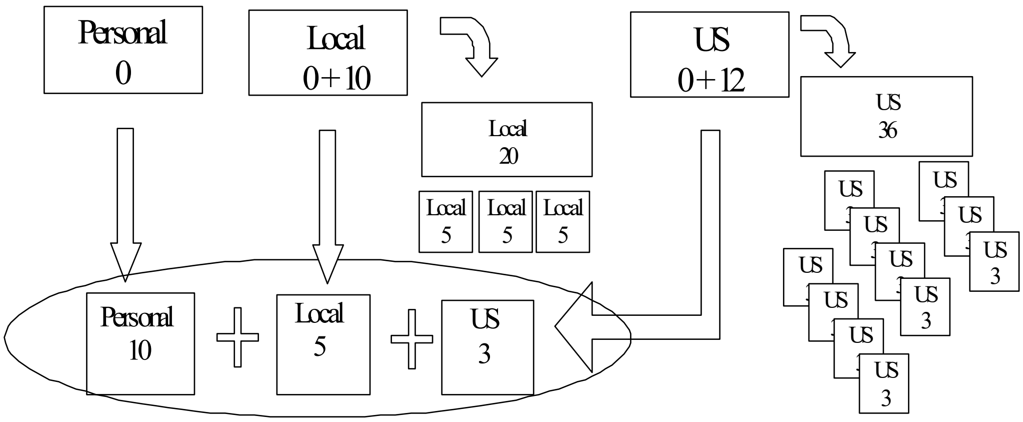

This is a little complicated, but is easy to figure out. First you would get 10 tokens from your "Personal" envelope. Second you would get 5 tokens from the <Local> share (the 10 tokens would be doubled and your share is 5). Finally you would get 3 tokens from the <COUNTRY NAME> share (the 12 tokens would be tripled to 36 and divided by 12, you get 3). Your total is 18 blue tokens and for that you would receive <$9.00≥. Note that every other member of your local group would get 5 tokens from the local share and 3 from their share of the <COUNTRY NAME> one, but the total tokens received by each of them would depend on the number of tokens allocated to the Personal envelope.

Here's another example (#2). Suppose you put your 10 blue tokens in the <COUNTRY NAME> envelope and 2 other tokens were put into the <COUNTRY NAME> envelope. No tokens were put into the local envelopes. How much would you receive? Go ahead and write on your example if you would like.

Example 2

You put 0 blue tokens in your "Personal" envelope. Others put 0 blue tokens in their <Local> envelopes. Finally, 12 blue tokens are put in the "US" envelopes.

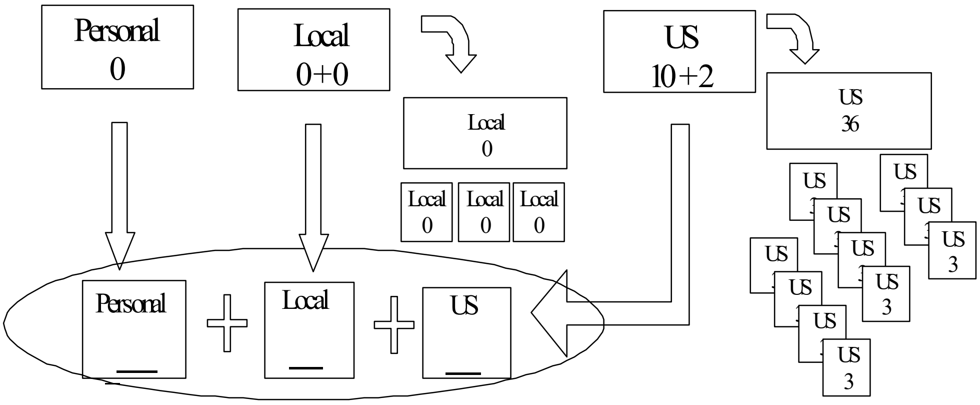

Pause and wait for participant to come up with an answer…

You would get nothing from your "Personal" envelope. Because no one else put anything into their <Local> envelopes you would get no share from that. Finally, the 12 blue tokens in the <COUNTRY NAME> envelopes would be tripled and your share would be 3 tokens. You would receive <$1.50>.

In the final example (#3) suppose you put no blue tokens in your "Personal" envelope, you put 2 tokens in your local envelope and the other three people put 10 blue tokens in their <Local> envelopes for a total of 12, and you and the other 11 people put a total of 80 blue tokens into their <COUNTRY NAME> envelopes. How much would you receive?

Example 3

You put 0 blue tokens in your "Personal" envelope. You and the others put a total of 12 blue tokens in their <Local> envelopes. Finally, 80 blue tokens are put in the "US" envelopes.

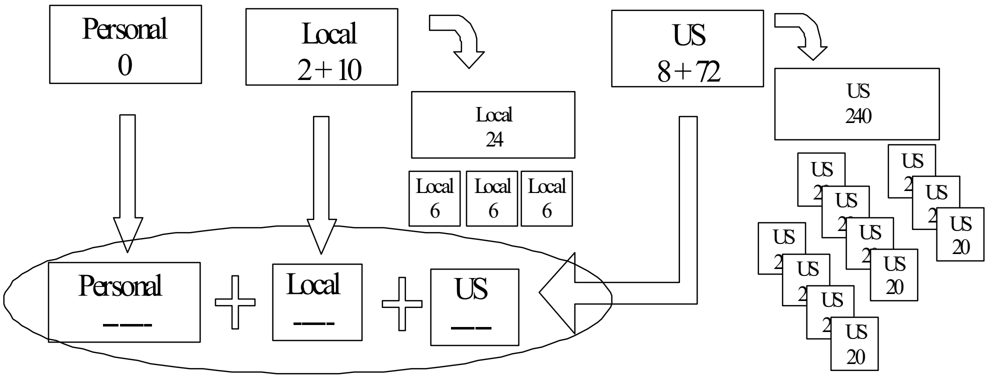

Wait for participant to come up with an answer…

Once again you would get nothing from your "Personal" envelope. The 12 tokens in the local envelopes would be doubled and your share (1/4) would be 6. In the <COUNTRY NAME> envelopes there are 80 blue tokens. These are tripled to 240 and your share (1/12th) is 20 blue tokens. For this you would receive a total of 26 tokens, worth <$13.00>. Again, does everyone understand how this result was obtained? These three examples show that what you get depends both on what you and everyone else does. Please take a minute to go through the examples.

Pause for one minute while people look over their materials.

Now it is time for you to make your decision. You can put any combination of tokens into the 3 envelopes. Remember that the blue tokens you put into your “Personal” envelope are yours and will not be divided among any others. Whatever you and the three other people from around this area put into the <Local> envelopes will be doubled. Each of you will get an equal share of that amount. Whatever you put into the <COUNTRY NAME> envelope will be tripled. You and 11 others will get an equal share of that amount. Please make your decision, DO NOT seal the envelopes and put the materials on top of your [desk/box]. My assistant will come around and collect your envelopes. The assistant will check to make certain you have put your ID number in the upper right corner of all three of your envelopes. When you are finished, please put all of your materials on top of your [box/desk].

The envelopes will be put into a box marked Decision 2. The assistant should double check each envelope to make certain that it has an ID number attached to it. If not, ask the subject to do it before the envelopes are placed in the box. Collect all materials.

The envelopes should be taken to a Core member of the team who is outside the room. The Core member should open the personal envelopes, enter and record the personal decision for each subject. The Core member should then open the local envelope, enter and record the number of blue tokens, check the group assignment, and calculate the share obtained by the Ss. The group total and the Ss share should be filled out on the decision slip of paper and the session log. Finally the <COUNTRY NAME> envelope should be opened, the appropriate information recorded and group totals calculated under the matching routine. The group total and the Ss share should be filled out on the decision slip and the session log.

Now that everyone’s decision has been made, the envelopes will be matched with other people and how much you money you receive will be calculated. It will take a while to do this. At the end of the session you will be given your payment in an envelope.

DECISION THREE.

You have now finished the second decision. The third decision is similar, but it has some changes, so please listen very carefully. In this decision you will have 10 yellow tokens and 3 envelopes. Once again you will be paid <$.50> for each yellow (colored) token.

In this decision you will be randomly mixed with different groups of people. First you will be mixed with three other people from this local area. It is very likely that it will be three people who are different from your first or second decision. The second group will be composed of 12 people. It will include the same three local people, plus two other groups of four people from countries around the world.

As I told you at the beginning, this research is being conducted by an international team. This study is being conducted with participants from all parts of the world, including Asia, Africa, Europe, and North and South America. The team is collecting decisions made by other people who are facing the same choices as you, and they are sending their results back to a central administrator who will tell us what different groups have decided to do. Your decision will be randomly mixed with what other people have done and this will determine your final payment. Your decisions will also affect the payments of the others in your group.

As with the second decision, the yellow tokens you put in your "Personal" envelope will be yours and not divided with anyone else. Second the yellow tokens you and the others put into the <Local> envelope will be doubled and you will get a 1/4 share from the local group. Finally you have a <WORLD> envelope. The yellow tokens that you and 11 other people put into those envelopes will be tripled. You will get an equal share of the tripled amount. The materials that my assistant will now hand out are similar to what you received for the previous decision. Please look these over and be sure that you understand before you begin.

Hand out Decision 3 materials.

Your task is to put your 10 tokens in the envelopes. You can put any combination of tokens into the 3 envelopes. You should have 10 yellow tokens (each of which are worth <$.50> to you and everyone else). Your tokens are in your "Personal" envelope. Please take them out and count them to make certain you have 10.

Please remove an ID sticker and put one on each of the three envelopes. Please do this now and make certain it is in the upper right corner.

Now it is time for you to make your decision. Remember that the yellow tokens you put into your "Personal" envelope are yours and will not be divided among any others. Whatever you and the three other people from around this area put into the <Local> envelopes will be doubled. Each of you will get an equal share of that amount. Whatever you put into the <WORLD> envelope will be tripled. You and 11 others will get an equal share of that amount. Please make your decision, DO NOT seal the envelopes and put the materials on top of your [desk/box]. My assistant will come around and collect your envelopes. The assistant will check to make certain you have put your ID number in the upper right corner of all three of your envelopes. When you are finished, please place all your materials on top of your [box/desk].

The envelopes will be put into a yellow box marked Decision 3. The assistant should double check each envelope to make certain that it has an ID number attached to it. If not, ask the subject to do it before the envelopes are placed in the box. Collect all materials.

The envelopes should be taken to a Core member of the team who is outside the room. The Core member should open the personal envelopes, enter and record the personal decision for each subject. The Core member should then open the local envelope, enter and record the number of yellow tokens; check the group assignment and calculate the share obtained by the S. The group total and the Ss share should be filled out on the decision slip and the session log sheet. Finally the world envelope should be opened, the appropriate information entered and recorded and group totals calculated under the matching routine. The group total and the Ss share should be filled out on the decision slip and the session log.

The core member then combines information from all three decisions for each participant and calculates their total payment, which is recorded on the decision slip for that participant (and on the session log sheet). The decision slip is put into an envelope, along with the total payment in money (rounded to the nearest whole number) and labeled with the participant ID number.

You have now finished making your decisions with others. Before you are given the payments from your decisions, I am going to have my assistant pass out a questionnaire. This questionnaire will help us get more information about the people who participated in the decisions. Your

questionnaire will have only your ID number. We will not know who you are and how you responded.   Please be as honest as you can with your answers.

The assistant passes out the questionnaire, with the expectancy measures first.

Please take the sticker off of your ID card and put it in the upper right corner of the first page of the questionnaire you have been handed. When you are finished, please place your questionnaire and pen on top of your [box/desk]. Once everyone is finished, we will wait while the payment calculations are being completed.

When all participants are finished they can be brought out one at a time, with their questionnaire, The questionnaire can serve as the "passport" out of the experiment;tt needs to have an ID on it for the subject to collect a pay envelope. The experimenter will then give the participant the payment envelope which matches the participant's ID#.

Participants should then count and verify that they have received the amount listed on the decision slip which is in the envelope, and the experimenter will fill in the participant's ID number on the log sheet, the amount received, and then put his/her initials next to it. The experimenter will then cover that particular line of the log sheet and the next participant will come in.

Decision 1

Example 1

You put 10 red tokens in your "Personal" envelope. Others put a total of 12 red tokens in their <Local> envelopes. Those <Local> envelope tokens are doubled and you get an equal share.

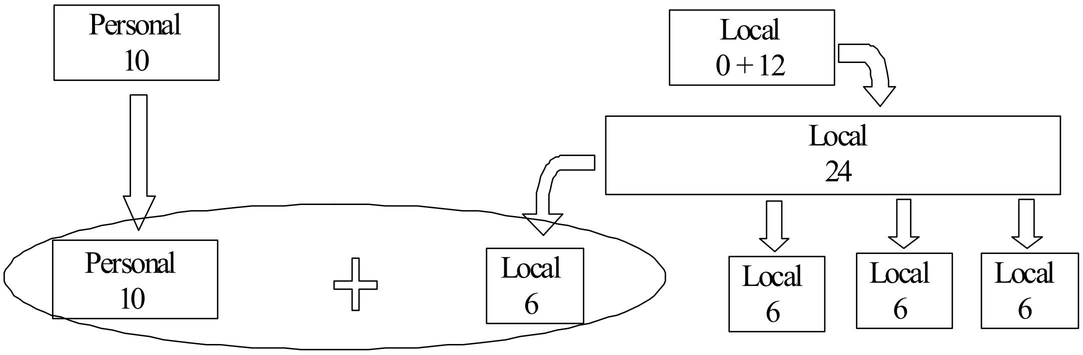

Example 2

You put 2 red tokens in your "Personal" envelope. Others put 0 red tokens in their <Local> envelopes.

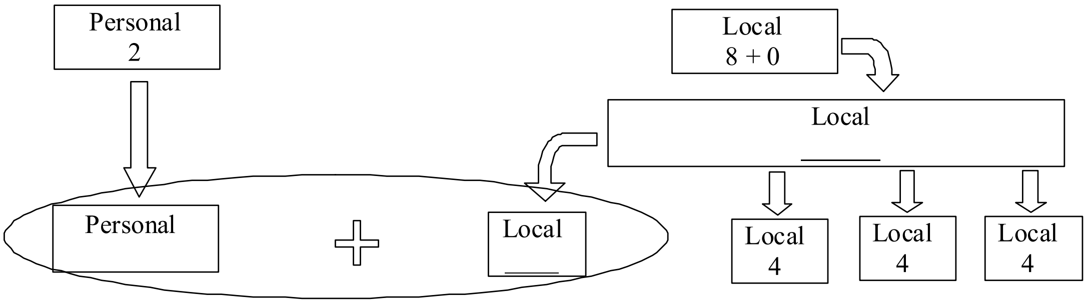

Example 3

You put 0 red tokens in your “Personal” envelope. Others put 30 red tokens in their <Local> envelopes.

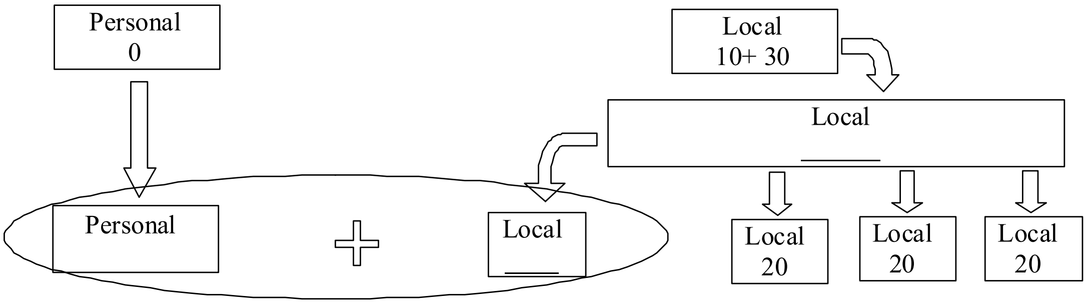

ID

Decision 1

Please check the correct box for each question.

1. What happens when a red token is put into the <Local> envelope?

- ❑ Nothing
- The token is cut in half
- The token is doubled
- ❑ The token is tripled

2. How many people, including you, are in the <Local> group?

- ❑ Two people
- Three people
- Four people
- ❑ Five people

3. Everyone gets an equal share of the <Local> envelope.

❑ True

❑ False

Decision 2

Example 1

You put 10 blue tokens in your “Personal” envelope. Others put a total of 10 blue tokens in their <Local> envelopes. Finally, 12 blue tokens are put in the “US” envelopes. Those “US” envelope tokens are tripled and you get an equal share.

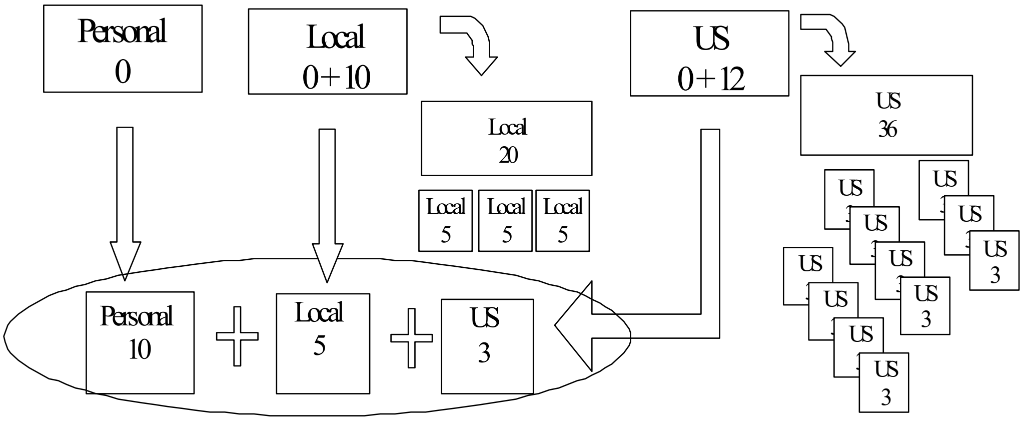

Example 2

You put 0 blue tokens in your “Personal” envelope. Others put 0 blue tokens in their <Local> envelopes. Finally, 12 blue tokens are put in the “US” envelopes.

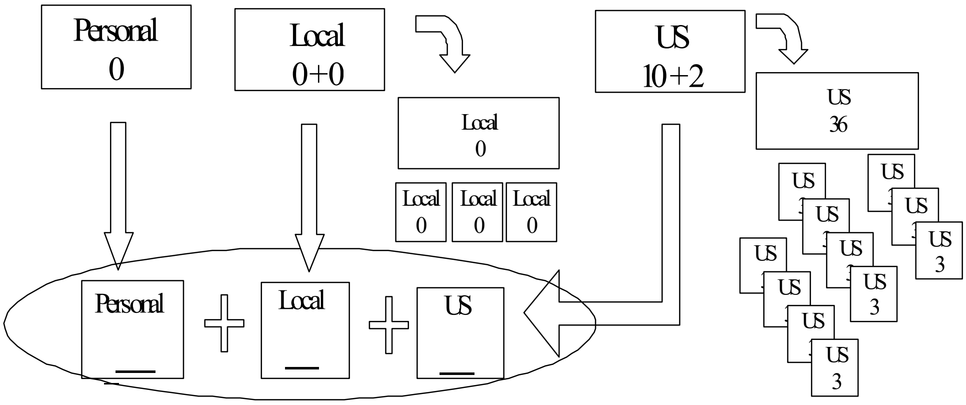

Example 3

You put 0 blue tokens in your “Personal” envelope. You and the others put a total of 12 blue tokens in their <Local> envelopes. Finally, 80 blue tokens are put in the “US” envelopes.

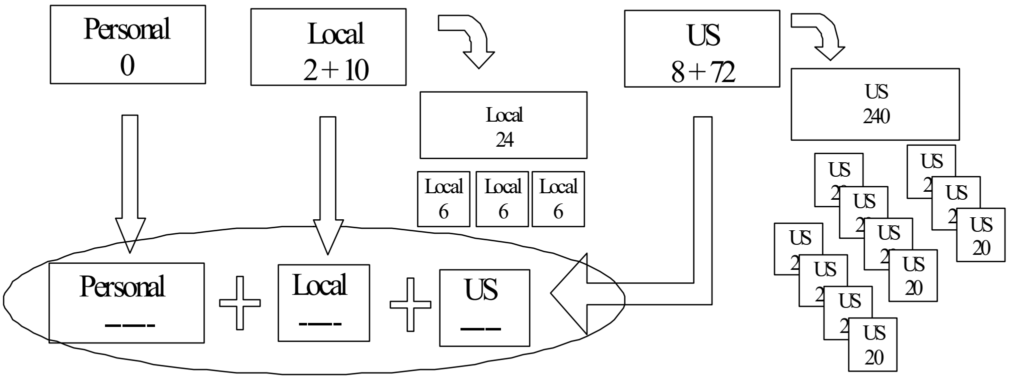

ID

Decision 1		
	Earnings from Decision 2	______________
Decision 2		
	Earnings from Decision 2	______________
Decision 3		
	Earnings from Decision 3	______________
Total Experiment Earnings in this Envelope	(rounded)	______________
Show-up Fee		______________
Total Amount of Money Claimed by Participant	(rounded)	______________

ID

In Decision 1 you had 10 red tokens. So did everyone else. You could put your tokens into your “Personal” envelope or into your <Local> envelope. The other three people in your local group could also choose to put tokens into their own personal envelope or into the <Local> envelope. Please answer the following questions.

1. How much do you think the other three people put into the <Local> envelopes in total (there is a maximum of 30 red tokens that they could put into them): ______________

2. How much money do you expect to receive from the <Local> envelope that will be returned to you: ___________

3. How much did you feel you were obliged to put money in the <Local> envelope, no matter what other people in the group did? (check one response)

____Not at all obliged

___ Somewhat obliged

__ Strongly obliged

In Decision 2 you had 10 blue tokens. So did everyone else. You could put your tokens into your “Personal” envelope, into your <Local> envelope or into the <*COUNTRY NAME*> envelope. Please answer the following questions.

1. How much do you think the other three people in your local group put into their <Local> envelopes (a maximum of 30 blue tokens that could be put into them): _______________

2. How much do you think the other 11 people in your <*COUNTRY NAME*> put into the “US” envelopes in total (a maximum of 110 blue tokens could be put into them): __________

3. How much did you feel you were obliged to put money in the <*COUNTRY NAME*>envelope, no matter what other people in the group did? (check one response)

___Not at all obliged

___Somewhat obliged

__Strongly obliged

In Decision 3 you had 10 yellow tokens. So did everyone else. You could put your tokens into your "Personal" envelope, into your <Local> envelope or into the "World" envelope. Please answer the following questions.

1. How much do you think was put into the <Local> envelopes by the other three people in your local group (a maximum of 30 yellow tokens that could be put into them): _______________

2. How much do you think was put into all of the "World" envelopes by the other 11 people in your group (a maximum of 110 yellow tokens could be put into them): __________

3. How much did you feel you were obliged to put money in the WORLD envelope, no matter what other people in the group did? (check one response)

___Not at all obliged

___Somewhat obliged

__Strongly obliged
