# Supplementary material for: Social identity mediates the positive effect of globalization on individual cooperation: Results from international experiments
Source: PLoS One. 2018 Dec 14;13(12):e0206819. doi: 10.1371/journal.pone.0206819 (PMC6294391; doi:10.1371/journal.pone.0206819)
Supplement: S1 Data — (ZIP) [file pone.0206819.s005.zip › Data/GCSI Questionnaire.pdf]

Questionnaire SIGC Project

{Classification Codes: [S]=Social globalisation; [C] = Cultural globalisation; [E]=Economic globalization; [GA]= Global Awareness; [SI]=Social Identity; [AMI]=Association Membership Index; [GAMI]=Global Association Membership Index }

Note: The whole version of the questionnaire can be found in Buchan et al. (2009: SOM). The questions reproduced below are those relevant for the analyses presented in this paper. Terms in italics were adapted to different localities / localities. The terms reported below were used in the community of Columbus, OH, US. The numbering of the question is the same as in the original questionnaire.

1. How often do you normally use the following products or services? Check one option.

		I own/have access to this product or service, and I use it:				I do not own/have access to this product or service.
		Every day	Every week	Less often	Never	
**[S]**	Internet	☐1	☐2	☐3	☐4	☐5
**[ECO]**	Credit card	☐1	☐2	☐3	☐4	☐5

2. If you use the following products or services, do you use them to contact people living in other parts of your country, or people living in other countries? Check all that apply.

			Local area	Other parts of my country	Other countries	Does not apply
**[S]**	a.	Landline phone	☐1	☐2	☐3	☐4
**[S]**	b.	Mobile phone	☐1	☐2	☐3	☐4
**[S]**	c.	Email	☐1	☐2	☐3	☐4
**[S]**	d.	Postal mail	☐1	☐2	☐3	☐4
**[E]**	e.	Fax machine	☐1	☐2	☐3	☐4

3. Consider the following geographical areas. How often do you travel, either for work or for vacation, in each of them? Check one.

		Every week	Every month	Every year	Less often	Never
	a. Within a national area (to other parts of my country besides my own locality).	☐1	☐2	☐3	☐4	☐5
**[C]**	b. To other countries within my continent	☐1	☐2	☐3	☐4	☐5
**[C]**	c. To other countries outside my continent	☐1	☐2	☐3	☐4	☐5

4. How concerned are you with the following issues? Check one.

		Not at all concerned	Slightly concerned	Concerned	Very concerned	I am not informed about this issue.
**[GA]**	a. Global warming	☐1	☐2	☐3	☐4	☐5
**[GA]**	b. The spread across the planet of potentially dangerous diseases (for example, HIV, SARS, bird flu)	☐1	☐2	☐3	☐4	☐5
**[GA]**	c. Making the action of International Criminal Courts of justice more effective	☐1	☐2	☐3	☐4	☐5
**[GA]**	d. The persistent gap between rich and poor people around the world	☐1	☐2	☐3	☐4	☐5

5. Have you taken part in the following activity?

		Yes	No
	a. Contributing to international aid efforts for natural disasters (for example, hurricanes in the US, earthquakes in Pakistan, tsunami)	☐1	☐2
	b. Contributing or participating in international aid efforts for poverty relief (for example, Live Eight)	☐1	☐2
**[C]**	c. Following international sport events (for example, Olympic games, soccer world cup)	☐1	☐2
**[C]**	d. Following international cultural events or international trade fairs	☐1	☐2

6. How often do you do the following activity? Check one.

		I own/have access to this product or service, and I use it:				I do not own/have access to this product or service.
		Every day	Every week	Less often	Never	
**[C]**	a. Watch a television program or a movie from a different country	☐1	☐2	☐3	☐4	☐5
**[C]**	b. Watch / listen to an international news source (CNN International, BBC World, *Euronews*)	☐1	☐2	☐3	☐4	☐5
**[C]**	c. Read an international news source (Time, The Economist, *Le Monde*)	☐1	☐2	☐3	☐4	☐5
**[C]**	d. Read an international magazine other than a news related publication, e.g. a style or sports magazine (Cosmopolitan, National Geographic, *Men's Health*)	☐1	☐2	☐3	☐4	☐5
**[C]**	e. Read a book written by an author from another country	☐1	☐2	☐3	☐4	☐5
**[C]**	f. Listen to music made by artists from other countries	☐1	☐2	☐3	☐4	☐5

9. **[E]** Do you work for a multinational or foreign-owned company?

Yes	No
☐1	☐2

10. **[E]** If you (or your household) own a car, where was it made? Check one (if you own more than one car, consider the one that you regularly use).

In my country	In a different country	I do not know where my car was made	I do not own a car
☐1	☐2	☐3	☐4

11a. Consider the following list; Are products or services that are from different parts of the world available in the area where you live?

	Yes	No	I don't know
A. Restaurants (e.g. *Japanese*, *Thai* restaurants)	☐1	☐2	☐3
B. Food and beverages (from supermarkets, shops or bars)	☐1	☐2	☐3
C. Clothing	☐1	☐2	☐3

11b. **[E]** If you have answered yes to the previous question, how often do you use such products or services?

	Every day	Every week	Less often	Never
a. Restaurants (e.g. *Japanese*, *Thai* restaurants)	☐1	☐2	☐3	☐4
b. Food and beverages (from supermarkets, shops or bars)	☐1	☐2	☐3	☐4
c. Clothing	☐1	☐2	☐3	☐4

12a. Consider the following list; Are products or services that are produced by multi-national companies - that is, those companies active in different parts of the world - available in the area where you live?

	Yes	No	I don't know.
A. Restaurants and cafes (e.g. Mc Donald's, Starbucks Coffee, *Pizza Hut*, *Taco Bell*)	☐1	☐2	☐3
B. Food and Beverages (e.g. Coca-Cola, Nestlé, *Dannon*)	☐1	☐2	☐3
C. Clothing (e.g. Nike, Zara, *Adidas*, *Levi's*)	☐1	☐2	☐3

12b. **[E]** If you have answered yes to the previous question, how often do you use such products?

	Every day	Every week	Less often	Never
A. Restaurants and cafes (e.g. Mc Donald's, Starbucks Coffee, *Pizza Hut*, *Taco Bell*)	☐1	☐2	☐3	☐4
B. Food and Beverages (e.g. Coca-Cola, *Nestle'*, *Dannon*)	☐1	☐2	☐3	☐4
C. Clothing (e.g. Nike, Zara, *Adidas*, *Levi's*)	☐1	☐2	☐3	☐4

14. Currently do you own any of the following?

		Yes	No
**[E]**	a. Foreign currencies	☐1	☐2
**[E]**	b. Bank deposit in another country	☐1	☐2
**[E]**	c. Some investment(s) in another country	☐1	☐2

15. **[S]** Besides your native tongue, how many other languages can you speak?

None	☐ 1
I can understand and can make myself understood in another language.	☐ 2
I am fluent in another language.	☐ 3
I am fluent in more than one other language.	☐ 4

16a. How many different immigrant communities live in the area where you live (for example, *Hmong immigrants*)?

None	☐ 1
Between 1 and 2	☐ 2
Between 3 and 4	☐ 3
More than that	☐ 4

17a. How many ethnic/racial groups different from yours live in the area where you live (for example, *White, Black/African Americans, Asian, Hispanic*)?

None	☐ 1
Between 1 and 2	☐ 2
Between 3 and 4	☐ 3
More than that	☐ 4
Several people (6 or more people)	☐ 3

21. **[SI]** How strongly do you feel attachment to your community in *Columbus*, in the *United States*, or to the world as a whole?

	Not attached at all			Very attached
a. Your local community	☐ 1	☐ 2	☐ 3	☐ 4
b. Your country	☐ 1	☐ 2	☐ 3	☐ 4
c. The world as a whole	☐ 1	☐ 2	☐ 3	☐ 4

22. **[SI]** How strongly do you define yourself as a member of your community in *Columbus*, in the *United States*, or of the world as a whole?

	Not at all			Very strongly
a. Your local community	☐ 1	☐ 2	☐ 3	☐ 4
b. Your country	☐ 1	☐ 2	☐ 3	☐ 4
c. The world as a whole	☐ 1	☐ 2	☐ 3	☐ 4

23. **[SI]** How close do you feel to other members of your community in *Columbus*, in the *United States*, or to the world as a whole?

	Not at all close			Very Close
a. Your local community	☐ 1	☐ 2	☐ 3	☐ 4
b. Your country	☐ 1	☐ 2	☐ 3	☐ 4
c. The world as a whole	☐ 1	☐ 2	☐ 3	☐ 4

25. **[AMI]** Please look carefully at the following list of voluntary organizations and activities. How would you describe your involvement with them? Check the one response that best applies for each type of activity or organization.

	I do not belong and do not follow their activities.	I do not belong but I sympathize with some of their activities.	I Belong
a. Social welfare services for elderly, *handicapped* or deprived people	☐1	☐2	☐3
b. Religious or church organizations	☐1	☐2	☐3
c. Education, arts, music or cultural activities	☐1	☐2	☐3
d. Labor unions	☐1	☐2	☐3
e. Political parties or groups	☐1	☐2	☐3
f. Poor countries development or human rights	☐1	☐2	☐3
g. Conservation, environmental, animal rights groups	☐1	☐2	☐3
h. Professional associations	☐1	☐2	☐3
i. Youth work (for example, *scouts, guides, youth clubs*, etc.)	☐1	☐2	☐3
j. Sports or recreation	☐1	☐2	☐3
k. Women's group	☐1	☐2	☐3
l. Peace movement	☐1	☐2	☐3
m. Voluntary organizations connected with health	☐1	☐2	☐3

26. **[GAMI]** If you have answered that you belong to some of the organizations listed above, please indicate whether the voluntary activity or organization is mainly active locally, nationally or internationally. Check as many responses as apply for each type of activity or organization.

	Local	National	International
a. Social welfare services for elderly, handicapped or deprived people	☐1	☐2	☐3
b. Religious or church organizations	☐1	☐2	☐3
c. Education, arts, music or cultural activities	☐1	☐2	☐3
d. Labor unions	☐1	☐2	☐3
e. Political parties or groups	☐1	☐2	☐3
f. Poor countries development or human rights	☐1	☐2	☐3
g. Conservation, environmental, animal rights groups	☐1	☐2	☐3
h. Professional associations	☐1	☐2	☐3
i. Youth work (for example, scouts, guides, youth clubs, etc.)	☐1	☐2	☐3
j. Sports or recreation	☐1	☐2	☐3
k. Women's group	☐1	☐2	☐3
l. Peace movement	☐1	☐2	☐3
m. Voluntary organizations connected with health	☐1	☐2	☐3

27. For each of the following statements, please state if you agree or disagree:

a. Our people are not perfect, but our culture is better than all others.

Completely disagree	Somewhat disagree	Somewhat agree	Completely agree
☐ 1	☐ 2	☐ 3	☐ 4

b. Our way of life needs to be protected against foreign influence.

Completely disagree	Somewhat disagree	Somewhat agree	Completely agree
☐ 1	☐ 2	☐ 3	☐ 4

c. We should restrict and control entry of people into our own country more than we do.

Completely disagree	Somewhat disagree	Somewhat agree	Completely agree
☐ 1	☐ 2	☐ 3	☐ 4

28. What do you think about each of the following: Has it been a very good thing, somewhat good, somewhat bad or very bad for you (and your family)?

28a. The world becoming more connected through greater economic trade and business ties?

Very Good	Somewhat Good	Somewhat Bad	Very Bad	Don't Know
☐1	☐2	☐3	☐4	☐5

28b. The world becoming more connected through faster communication and greater movements of people?

Very Good	Somewhat Good	Somewhat Bad	Very Bad	Don't Know
☐1	☐2	☐3	☐4	☐5

30. What is your sex?

Male	Female
☐1	☐2

31. In which year were you born? ____________________

32. What is the highest level of education you completed?

Grade School	*High School*	*Technical School*	*Bachelors Degree*	*Masters Degree*	*Doctoral Degree*
☐1	☐2	☐3	☐4	☐5	☐6

			Yes	No
**[S]**	33a.	Were you born in a country different than the US?	☐1	☐2
**[S]**	33b.	Were any of your parents born in a country different than *the US*?	☐1	☐2

35. What is your marital status?

Single	Married	Divorced/ Separated	Widowed	Living with partner
☐1	☐2	☐3	☐4	☐5

36. What is your current employment situation?

Full-time employed	☐1
Part-time employed	☐2
Self-employed	☐3
Retired/Pensioned	☐4
Housewife/husband not otherwise employed	☐5
Student	☐6
Unemployed	☐7
Other (Please specify) ____________________________	☐8

37. In which profession/occupation do you or did you work? If more than one job, the main job? What is/was your job there?

____
_

38. Here is a scale of incomes. We would like to know in what group your household is, counting all wages, salaries, pensions and other incomes that come in. Just check the group your household falls into, before taxes and other deductions.

$0-9,999	*10,000-14,999*	*15,000-24,999*	*25,000-34,999*	*35,000-49,999*	*50,000-74,999*	*75,000-99,999*	*100,000-149,999*	*150,000-199,999*	*Over 200,000*
☐1	☐2	☐3	☐4	☐5	☐6	☐7	☐8	☐9	☐10

Notes to Local Collaborators for Adapting Questionnaire to Local Environment

The present questionnaire is the version that was tailored for a US location (Portage, WI) for a pilot test. Hence, several questions require adaptation to the country/locality where the research is conducted. In particular, all items highlighted in yellow require some change. Some of these are obvious, e.g. substituting the name of your country for the US. Some others are less obvious. In particular, this is the case for the questions that provide examples for certain items, such as satellite channels, newspapers, *etc*. Normally, there will be 3 examples for each of these items, which include (a) the most widespread item worldwide; (b) the most widespread item worldwide coming from a different continent than the first one (this is to avoid listing mainly made-in-US brands); (c) another widespread item in the country's region. By region we mean the continental or sub-continental geographical entity to which a country belongs (e.g. North-America; Latin America; Sub-Saharian Africa; Europe; Asia; former Soviet Republics; Middle East). The questionnaire generally provides examples for (a) and (b), but identifying (c) is left to the local researcher's expertise. The following are suggestions derived from a variety of sources (PEW Global Attitudes Project: 06.03.03, Views of a Changing World, Summer 2002 44-Nation Survey http://pewglobal.org/datasets/, UNCTAD databases, internet):

Q6b: ask "CNN International, BBC World" in every country. Add a third example in each country as per Q60 in Pew survey, or local researcher's advice (e.g. Al Jazeera for Iran; Euronews for Russia and Europe; another region-wide channel for the US; DSTV for South Africa).
Q6c: Ask "Time, the Economist" in every country; provide a third example as per local researcher's advice (e.g. International Herald Tribune for non-English speaking countries; Le Monde for English-speaking countries).
Q6d: Ask "Cosmpolitan, National Geographic" in every country. Then ask another magazine widespread in the region (e.g. Men's Health, or Reader's Digest). You can find other examples at http://www.allyoucanread.com/Top20/ (Note: ignore adult magazines).
Q11a,b: provide example as per local researcher's advice

Q12a: ask “Mc Donald’s, Starbucks Coffee” in every country (They are the ‘icons’ of globalisation). For the other two items, rely on the local collaborator’s advice. The third and fourth items may be Pizza Hut or KFC, which may nevertheless be absent in some countries. Try whenever possible to ensure varieties in the type of food provided in the restaurants making up the examples. It is very difficult to find non-US food chains.

Q12b: Ask “Coca-Cola, Nestlé” in every country. Third example as per local researcher’s advice. Possible (non-US) items are Dannon (for US) or Danone, and Nescafè.

Q12c Ask Nike and Zara in each country. Third example as per local researcher’s advice. This may for instance be Adidas for the US (a German brand), or Levi’s for other countries.

NB: You may find other examples of transnational corporations at the Businessweek Top 100 global brands scoreboard (http://bwnt.businessweek.com/brand/2005/) or on the UNCTAD ranking of TNCs (http://www.unctad.org/Templates/Page.asp?intItemID=2443&lang=1)

Q32: as per Q84 (Pew), or local researcher’s advice

Q36: as per local researcher’s advice.

Q38: provide income categories considering the deciles of the income distribution in your country. Do not compute such categories at the current exchange rate between your currency and the USD, as that will probably make comparisons impossible. Use conversions via purchasing power parity and the consensus of local experts.
