# Supplementary material for: Social identity mediates the positive effect of globalization on individual cooperation: Results from international experiments
Source: PLoS One. 2018 Dec 14;13(12):e0206819. doi: 10.1371/journal.pone.0206819 (PMC6294391; doi:10.1371/journal.pone.0206819)
Supplement: S1 Data — (ZIP) [file pone.0206819.s005.zip › Data/READ ME.pdf]

INSTRUCTIONS ON HOW TO USE DATASET FOR “SOCIAL IDENTITY, GLOBALIZATION, COOPERATION” PROJECT AND REPLICATE ANALYSES INCLUDED IN PAPER PUBLISHED IN PLOS1

By Gianluca Grimalda

- The file “GCSI_Data.csv” contains raw data prior to construction of indexes.
- The numeric value attributed to each variable is indicated in the Questionnaire (see files “GCSI Questionnaire.pdf” and “GCSI data-CODEBOOK.pdf”).
- The file "GCSI_var_construction.txt" reports codes to construct the indexes and to create additional variables.
- The file "GCSI_Econometric_Mediation_analysis.txt" includes codes to replicate the analyses reported in the paper “Social identity mediates the positive effect of globalization on individual cooperation: Results from international experiments”, by Gianluca Grimalda, Nancy Buchan, Marilynn Brewer published in PLOS 1.
- Running the file "GCSI_Econometric_Mediation_analysis.txt" automatically launches the "SIGC_var_construction" code file, so there is no need to run the latter prior to the former.
- Running the file "GCSI_Econometric_Mediation_analysis.txt" includes codes for the econometric analyses and mediation tests. Output of such analyses is executed and written in a file that will be stored in the same folder where data and codes are stored.
- For the correct operation of the codes, it is necessary to place all the files in the same folder. The "change directory" (cd command) written at the beginning of the codes files should point to the folder where files are stored.
- The codes have been developed in Stata©.
