# Supplementary material for: Social identity mediates the positive effect of globalization on individual cooperation: Results from international experiments
Source: PLoS One. 2018 Dec 14;13(12):e0206819. doi: 10.1371/journal.pone.0206819 (PMC6294391; doi:10.1371/journal.pone.0206819)
Supplement: S1 Data — (ZIP) [file pone.0206819.s005.zip › Data/SIGC data-CODEBOOK.pdf]

id	Original ID		
new_id	A 4-digit number identifying subjects. The first digit identifies the country as per the codes for variable country below. The following three digits coincide with the subject's original ID (adjustments were made when necessary).		
country	1=Iran; 2=Russia; 3=SA; 4=US; 5=Argentina; 6=Italy		
location	Identifies locations where research was run for each country		
session	Identifies session for each country		
local1	Contribution to Local account in Decision 1		
local2	Contribution to Local account in Decision 2		
nation2	Contribution to National account in Decision 3		
local3	Contribution to Local account in Decision 3		
world3	Contribution to World account in Decision 3		
out2	nation2-local2		
out3	world3-local3		
coop2	nation2 + local2		
coop3	world3 + local3		
w3_n2	worold3-nation2		
l3_l2	local3-local2		
c3_c2	coop3-coop2		
o3_o2	out3-out2		
l1_trust	Expected allocations to local account by other group members in Decision 1		
l2_trust	Expected allocations to local account by other group members in Decision 2		
n2_trust	Expected allocations to national account by other group members in Decision 2		
l3_trust	Expected allocations to local account by other group members in Decision 3		
w3_trust	Expected allocations to world account by other group members in Decision 3		
obb_l	Obbligation measure: 1= 'Not at all'; 2='Somewhat'; 3='Strongly' obliged to allocating tokens into local account in D1		
obb_n	Obbligation measure: 1= 'Not at all'; 2='Somewhat'; 3='Strongly' obliged to allocating tokens into national account in D2		
obb_w	Obbligation measure: 1= 'Not at all'; 2='Somewhat'; 3='Strongly' obliged to allocating tokens into world account in D3		
mistakes	When available, data on number of mistakes in comprehension check. This ranges from 0 to 3.		
pho	As per answers to Q	1a	
mob	As per answers to Q	1b	
internet	As per answers to Q	1c	
email	As per answers to Q	1d	
cred	As per answers to Q	1e	
post	As per answers to Q	1f	
tv	As per answers to Q	1g	
tvs	As per answers to Q	1h	
radio	As per answers to Q	1i	
fax	As per answers to Q	1j	
pholoc	As per answers to Q	2a	Variable equals 1 if participant selects option "Local"
phonat	As per answers to Q	2a	Variable equals 1 if participant selects option "National"
phoglob	As per answers to Q	2a	Variable equals 1 if participant selects option "World"
phonot	As per answers to Q	2a	Variable equals 1 if participant selects option "Does not apply"
mobloc	As per answers to Q	2b	Variable equals 1 if participant selects option "Local"
mobnat	As per answers to Q	2b	Variable equals 1 if participant selects option "National"
mobglob	As per answers to Q	2b	Variable equals 1 if participant selects option "World"
mobnot	As per answers to Q	2b	Variable equals 1 if participant selects option "Does not apply"
emailloc	As per answers to Q	2c	Variable equals 1 if participant selects option "Local"
emailnat	As per answers to Q	2c	Variable equals 1 if participant selects option "National"
emailglo	As per answers to Q	2c	Variable equals 1 if participant selects option "World"
emailnot	As per answers to Q	2c	Variable equals 1 if participant selects option "Does not apply"
postloc	As per answers to Q	2d	Variable equals 1 if participant selects option "Local"
postnat	As per answers to Q	2d	Variable equals 1 if participant selects option "National"
postglob	As per answers to Q	2d	Variable equals 1 if participant selects option "World"

postnot	As per answers to Q	2d	Variable equals 1 if participant selects option "Does not apply"
faxloc	As per answers to Q	2e	Variable equals 1 if participant selects option "Local"
faxnat	As per answers to Q	2e	Variable equals 1 if participant selects option "National"
faxglob	As per answers to Q	2e	Variable equals 1 if participant selects option "World"
faxnot	As per answers to Q	2e	Variable equals 1 if participant selects option "Does not apply"
travnat	As per answers to Q	3a	
travcon	As per answers to Q	3b	
travglob	As per answers to Q	3c	
globwarm	As per answers to Q	4a	
disease	As per answers to Q	4b	
court	As per answers to Q	4c	
gap	As per answers to Q	4d	
aiddis	As per answers to Q	5a	
aidpov	As per answers to Q	5b	
sport	As per answers to Q	5c	
intevent	As per answers to Q	5d	
w_tv	As per answers to Q	6a	
w_news	As per answers to Q	6b	
r_news	As per answers to Q	6c	
r_mag	As per answers to Q	6d	
book	As per answers to Q	6e	
music	As per answers to Q	6f	
un	As per answers to Q	7a	
mnc	As per answers to Q	7b	
intorg	As per answers to Q	7c	
ngo	As per answers to Q	7d	
contrun	As per answers to Q	8a	
intagr	As per answers to Q	8b	
multcomp	As per answers to Q	9	
car	As per answers to Q	10	
rest	As per answers to Q	11aa	
food	As per answers to Q	11ab	
clot	As per answers to Q	11ac	
resto	As per answers to Q	11ba	
foodo	As per answers to Q	11bb	
cloto	As per answers to Q	11bc	
restmnc	As per answers to Q	12aa	
foodmnc	As per answers to Q	12ab	
clothmnc	As per answers to Q	12ac	
restmnco	As per answers to Q	12ba	
foodmnco	As per answers to Q	12bb	
clotmnco	As per answers to Q	12bc	
forprod	As per answers to Q	13	
forcurr	As per answers to Q	14a	
bank	As per answers to Q	14b	
invest	As per answers to Q	14c	
forlang	As per answers to Q	15	
forimm	As per answers to Q	16b	
friendfo	As per answers to Q	16b	
ethgroup	As per answers to Q	17a	
friendet	As per answers to Q	17b	
trust	As per answers to Q	18	
fair	As per answers to Q	19	

helpful	As per answers to Q	20	
a_local	As per answers to Q	21a	
a_nat	As per answers to Q	21b	
a_global	As per answers to Q	21c	
m_local	As per answers to Q	22a	
m_nat	As per answers to Q	22b	
m_global	As per answers to Q	22c	
c_local	As per answers to Q	23a	
c_nat	As per answers to Q	23b	
c_global	As per answers to Q	23c	
l_local	As per answers to Q	24a	
l_nat	As per answers to Q	24b	
l_global	As per answers to Q	24c	
socwel	As per answers to Q	25a	
church	As per answers to Q	25b	
educ	As per answers to Q	25c	
labor	As per answers to Q	25d	
polit	As per answers to Q	25e	
dcs	As per answers to Q	25f	
conserv	As per answers to Q	25g	
profass	As per answers to Q	25h	
youth	As per answers to Q	25i	
sports	As per answers to Q	25j	
women	As per answers to Q	25k	
peace	As per answers to Q	25l	
health	As per answers to Q	25m	
socwell	As per answers to Q	26aL	Variable equals 1 if participant selects option "Local"
socweln	As per answers to Q	26aN	Variable equals 1 if participant selects option "National"
socwelg	As per answers to Q	26aG	Variable equals 1 if participant selects option "World"
churchl	As per answers to Q	26bL	Variable equals 1 if participant selects option "Local"
churchn	As per answers to Q	26bN	Variable equals 1 if participant selects option "National"
churchg	As per answers to Q	26bG	Variable equals 1 if participant selects option "World"
educl	As per answers to Q	26cL	Variable equals 1 if participant selects option "Local"
educn	As per answers to Q	26cN	Variable equals 1 if participant selects option "National"
educg	As per answers to Q	26cG	Variable equals 1 if participant selects option "World"
laborl	As per answers to Q	26dL	Variable equals 1 if participant selects option "Local"
laborn	As per answers to Q	26dN	Variable equals 1 if participant selects option "National"
laborg	As per answers to Q	26dG	Variable equals 1 if participant selects option "World"
politl	As per answers to Q	26eL	Variable equals 1 if participant selects option "Local"
politn	As per answers to Q	26eN	Variable equals 1 if participant selects option "National"
politg	As per answers to Q	26eG	Variable equals 1 if participant selects option "World"
dcsl	As per answers to Q	26fL	Variable equals 1 if participant selects option "Local"
dcsn	As per answers to Q	26fN	Variable equals 1 if participant selects option "National"
dcsg	As per answers to Q	26fG	Variable equals 1 if participant selects option "World"
conservl	As per answers to Q	26gL	Variable equals 1 if participant selects option "Local"
conservn	As per answers to Q	26gN	Variable equals 1 if participant selects option "National"
conservg	As per answers to Q	26gG	Variable equals 1 if participant selects option "World"
profassl	As per answers to Q	26hL	Variable equals 1 if participant selects option "Local"
profassn	As per answers to Q	26hN	Variable equals 1 if participant selects option "National"
profassg	As per answers to Q	26hG	Variable equals 1 if participant selects option "World"
youthl	As per answers to Q	26iL	Variable equals 1 if participant selects option "Local"
youthn	As per answers to Q	26iN	Variable equals 1 if participant selects option "National"
youthg	As per answers to Q	26iG	Variable equals 1 if participant selects option "World"

sportsl	As per answers to Q	26jL	Variable equals 1 if participant selects option "Local"
sportsn	As per answers to Q	26jN	Variable equals 1 if participant selects option "National"
sportsg	As per answers to Q	26jG	Variable equals 1 if participant selects option "World"
womenl	As per answers to Q	26kL	Variable equals 1 if participant selects option "Local"
womenn	As per answers to Q	26kN	Variable equals 1 if participant selects option "National"
womeng	As per answers to Q	26kG	Variable equals 1 if participant selects option "World"
peacel	As per answers to Q	26lL	Variable equals 1 if participant selects option "Local"
peacen	As per answers to Q	26lN	Variable equals 1 if participant selects option "National"
peaceg	As per answers to Q	26lG	Variable equals 1 if participant selects option "World"
healthl	As per answers to Q	26mL	Variable equals 1 if participant selects option "Local"
healthn	As per answers to Q	26mN	Variable equals 1 if participant selects option "National"
healthg	As per answers to Q	26mG	Variable equals 1 if participant selects option "World"
culture	As per answers to Q	27a	
waylife	As per answers to Q	27b	
entry	As per answers to Q	27c	
conn_eb	As per answers to Q	28a	
conn_cm	As per answers to Q	28b	
satlife	As per answers to Q	29	
gender	As per answers to Q	30	
year	As per answers to Q	31	
educat	As per answers to Q	32	
country1	As per answers to Q	33a	
count_p	As per answers to Q	33b	
famres	As per answers to Q	33c	
youres	As per answers to Q	33d	
ethn	As per answers to Q	34	
status	As per answers to Q	35	
income	As per answers to Q	38	
arg_doll	As per answers to Q	14a	
arg_euro	As per answers to Q	14a	
arg_othe	As per answers to Q	14a	
employ_o	As per answers to Q	36b	
csgr_ind	Country Level Globalisation index		
gdp	Country Gross Domestic Product as of 2007		
